# Myelin Maintenance and Addition Regulate Synaptic Plasticity in the Adult Mouse Cortex

**DOI:** 10.1002/glia.70239

**Published:** 2026-10-11

**Authors:** Renee E. Pepper, Kalina Makowiecki, Kimberley A. Pitman, Raphael Ricci, Angela L. Nicoli, Phuong Tram Nguyen, Catherine A. Blizzard, Ben Emery, Carlie L. Cullen, Kaylene M. Young

**Affiliations:** ^1^ Menzies Institute for Medical Research University of Tasmania Hobart Australia; ^2^ Oregon Health and Science University Portland Oregon USA; ^3^ Mater Research Institute The University of Queensland Woolloongabba Queensland Australia

**Keywords:** demyelination, dendrite, excitation, glutamate, mEPSC, myelin, myelin regulatory factor (Myrf), oligodendrocyte, plasticity, spine

## Abstract

Myelin has the capacity to affect synapse number or plasticity across the lifespan. We report that disrupting oligodendrocyte (OL) and myelin maintenance from P57 (*Plp‐CreER* ^*T2*^:: *Myrf* ^*fl/fl*^ mice) induces myelin loss and impairs action potential conduction and gross motor performance. By studying sparsely labeled layer V pyramidal neurons in the primary motor cortex of P57 + 60 mice, we determined that apical and basal dendritic spine densities were normal; however, more were large, stable mushroom spines that generated larger amplitude miniature excitatory postsynaptic currents. When we instead prevented OL and myelin addition from P57 (*Pdgfrα‐CreER:: Myrf* ^*fl/fl*^ mice), action potential conduction velocity, gross motor performance, and spine densities were normal; however, basal dendritic spine morphology was changed. Without adult myelination, the basal dendrites of P57 + 60 mice retained the morphological characteristics of dendrites in P57 + 0 mice, which have a higher proportion of thin, plastic spines. These data indicate that existing myelin imposes limits on the proportion of spines that become mushroom spines, allowing neurons to remain plastic, while adult myelination is required for spine stabilization within the basal but not apical dendritic compartment. These data show that myelin regulates synaptic plasticity within the motor circuit and offer a potential mechanism by which myelination can influence motor skill learning and/or recall.

## Graphical abstract

We report that existing myelin allows neurons to remain plastic throughout life, while new myelin progressively stabilises synapses along the basal but not apical dendrites of layer V pyramidal neurons in the motor cortex.

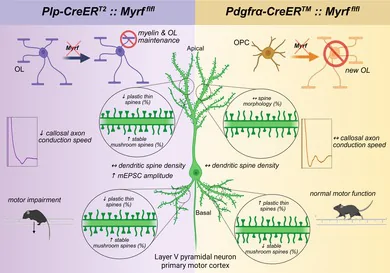

## 1 Introduction

The motor cortex (MC) supports the acquisition and execution of dexterous motor skills (Hayashi‐Takagi et al. 2015; Kida et al. 2023; Li et al. 2017). Within the MC, layer V pyramidal neurons extend their apical tuft dendrites dorsally to receive inputs from layer I but extend their basal dendrites to receive inputs from within layer V (Oswald et al. 2013; Spruston 2008). The apical and basal dendrites operate as functionally distinct neuronal compartments (Wright et al. 2025), but both receive and integrate sensory, state, and motor information from myelinated cortico‐cortical or thalamo‐cortical neurons to determine MC output (Geng et al. 2022; Hwang et al. 2022; Li et al. 2017; Neske and Cardin 2025; Ramot et al. 2025; Xu et al. 2009). Spines protruding from each dendrite correspond to the postsynaptic compartments of glutamatergic synapses. Spines transition from small, thin spines into large mushroom‐shaped spines as they are stabilized and strengthened through long‐term potentiation (Holtmaat et al. 2005; Matsuzaki et al. 2004). The acquisition and execution of learned and voluntary movements requires synaptic plasticity in the MC, including the plasticity of excitatory synapses onto layer V pyramidal neurons (Albarran et al. 2021; Hayashi‐Takagi et al. 2015; Hwang et al. 2022; Kida et al. 2023; Kida et al. 2024; Li et al. 2017; Ramot et al. 2025; Xu et al. 2009). As developmental myelination was recently shown to decrease the density of dendritic spines on layer V pyramidal neurons in the visual cortex (Xin et al. 2024), we aimed to determine whether existing myelin or new myelin are critical regulators of synapse density or plasticity in the adult mouse MC.

Oligodendrocytes (OLs) myelinate excitatory (Hughes et al. 2018; Tomassy et al. 2014; Young et al. 2013) and inhibitory (Micheva et al. 2016; Stedehouder et al. 2018; Stedehouder et al. 2017) neurons within the adult central nervous system (CNS), including neurons critical for motor learning and the precise execution of learned and voluntary movements (Bacmeister et al. 2020; Gibson et al. 2014; McKenzie et al. 2014; Xiao et al. 2016). Myelination supports the rapid and saltatory conduction of action potentials (Barrett and Barrett 1982; Freeman et al. 2016; Lubetzki et al. 2020) and promotes axon health through the provision of metabolites for local ATP synthesis (Brown et al. 2003; Meyer et al. 2018; Saab et al. 2016) and the release of exosomes to promote active transport (Fruhbeis et al. 2020). In development, cortical myelination coincides with the closure of critical periods when the cortical circuitry, including the cortical synaptic connections can change rapidly in response to environmental input (Fletcher et al. 2021; Reh et al. 2020). Cortical myelination can act as a physical and molecular plasticity brake, as myelination can limit neurite reorganization (Boghdadi et al. 2018), modulate neurotransmitter release (Chen et al. 2015; Maheras et al. 2018; Roy et al. 2007), reduce synaptogenesis (Zemmar et al. 2018) and synapse turnover (Xin et al. 2024), and regulate the intrinsic excitability of axons (Hamada and Kole 2015). As OLs are long‐lived cells (Hughes et al. 2018; Tripathi et al. 2017), the myelin they lay down and subsequently maintain has the potential to regulate synaptic plasticity throughout life.

While the majority of OLs present in the mature CNS correspond to those generated during development (Tripathi et al. 2017; Yeung et al. 2014), new OLs and myelin are generated throughout life (Dimou et al. 2008; Hill et al. 2018; Hughes et al. 2018; Kang et al. 2010; Rivers et al. 2008; Yeung et al. 2014; Young et al. 2013) and support the long‐term recollection of acquired skills and memories (McKenzie et al. 2014; Pan et al. 2020; Steadman et al. 2020; Xiao et al. 2016). As memory formation and recall are encoded through synaptic plasticity (Hayashi‐Takagi et al. 2015; Nabavi et al. 2014; Xu et al. 2009), this suggests that adult myelination has the capacity to alter synapse number or structure. While increasing the activity of layer V pyramidal neurons can increase new OL and myelin addition to the deep layers of the ipsilateral MC and corpus callosum (CC) (Gibson et al. 2014), it is unclear whether adult myelination modulates the number or stability of excitatory inputs received by the layer V MC output neurons.

Herein, we investigate the capacity for existing and new myelin to regulate synaptic plasticity in the MC under basal conditions. We show that genetically preventing OL and myelin maintenance in adulthood slows compound action potential conduction, impairs motor performance, and significantly increases the proportion of spines that are stable mushroom spines along the apical and basal dendrites of layer V MC neurons. We find that OLs and myelin must be maintained for neurons to balance synaptic plasticity with stabilization. By contrast, preventing the addition of new OLs did not alter compound action potential conduction or the execution of developmentally acquired motor skills. However, it significantly impaired the stabilization of excitatory synapses on the basal but not apical dendrites of layer V neurons. Without adult myelination, the basal dendrites of P57 + 60 mice appeared “frozen” in a P57 + 0 state, with an increased proportion of transient thin spines and fewer stable mushroom spines, suggesting that adult myelination supports the selective and cumulative stabilization of synapses across the lifespan.

## 2 Methods

### 2.1 Resource Availability

Resource information is provided in Table S1. Requests for resources, reagents, or data should be directed to the corresponding author (kaylene.young@utas.edu.au).

### 2.2 Transgenic Mice

All animal experiments were approved by the University of Tasmania Animal Ethics Committee (A0013471 and A0016151) and were carried out in accordance with the Australian code of practice for the care and use of animals for scientific purposes. *Plp‐CreER* ^*T2*^ (Doerflinger et al. 2003), *Pdgfrα‐CreER* (Kang et al. 2010), *Rosa26‐YFP* (Srinivas et al. 2001), and *Thy1‐YFPH* (Feng et al. 2000) transgenic mice were purchased from Jackson Laboratories, and *Myrf* ^*fl/fl*^ transgenic mice (Emery et al. 2009) were provided by Ben Emery (Oregon Health and Science University; see Table S1 for further information). Each was maintained as a live colony on the C57BL/6 background at the University of Tasmania and intercrossed to generate experimental mice that were weaned > P35 to ensure normal myelin development. Mice were group housed (2–5 per cage) in same‐sex groups in individually ventilated Optimice cages on a 12‐h light/dark cycle with free access to food and water. Mice bred for behavioral experiments were handled for ~5 min daily for 2 weeks prior to commencing experiments.

### 2.3 Genomic DNA Extraction and PCR Genotyping

Genomic DNA was extracted from mouse ear tissue biopsies, and 50–100 ng of genomic DNA was used to perform polymerase chain reaction (PCR) to detect the relevant transgenes as previously described (Auderset et al. 2020). The following primers were used to detect the *Rosa26‐YFP* transgene: Rosa26 wildtype 5′ AAAGT CGCTC TGAGT TGTTAT; Rosa26 wildtype 3′ GGAGC GGGAG AAATG GATATG and Rosa26 YFP 5′ GCGAAGAGTTTGTCCTCAACC [94°C for 4 min; 37 cycles of 94°C for 30 s, 60°C for 45 s and 72°C for 1 min, and a final 72°C for 10 min]. *Pdgfrα‐CreER* and *Plp‐CreER* ^*T2*^ mice were genotyped using primers that bind to *Cre recombinase*: Cre 5′ CAGGT CTCAG GAGCT ATGTC CAATT TACTG ACCGTA and Cre 3′ GGTGT TATAA GCAAT CCCCA GAA [94°C for 4 min, 34 cycles of 94°C for 30 s, 62°C for 45 s and 72°C for 1 min, and a final 10 min at 72°C]. The *Myrf floxed* gene was detected with Myrf 5′ AGGAG TGTTG TGGGA AGTGG and Myrf 3′ CCCAG GCTGA AGATG GAATA [94°C for 4 min, 34 cycles of 94°C for 30 s, 62°C for 45 s and 72°C for 1 min, and a final 10 min at 72°C]. Mice carrying the *Thy1‐YFPH* transgene were genotyped by RT‐PCR (Transnetyx).

### 2.4 Tamoxifen Administration and Tissue Preparation

Tamoxifen (Sigma‐Aldrich) was dissolved to 40 mg/mL in corn oil in a sonicating water bath (≥ 1 h until dissolved). For conditional gene deletion and lineage tracing, postnatal day (P)57 mice received 300 mg/kg of tamoxifen by oral gavage, daily for 4 consecutive days. Mice were analyzed at various time points following tamoxifen administration, and the number of days is indicated in the text using the nomenclature P57 + (days).

For tissue collection, mice were terminally anesthetized with a 180 mg/kg intraperitoneal (i.p.) injection of sodium pentobarbitone for transcardial perfusion at ~9 mL/min with 4% paraformaldehyde (PFA; Sigma‐Aldrich) (w/v) in phosphate‐buffered saline (PBS). Brains were removed post‐mortem, and the 2 mm coronal brain slices, generated using a brain matrix (Kent Scientific), were immersion‐fixed in 4% PFA (w/v) in PBS for 90 min at ~21°C. Tissue was cryoprotected by immersion in 20% sucrose (Sigma‐Aldrich) (w/v) in PBS overnight at 4°C prior to embedding in optimal cutting temperature cryomatrix (Thermo Scientific) and storage at −80°C.

### 2.5 Immunohistochemistry, Confocal Microscopy and Quantification

30 μm coronal brain cryosections were generated for immunohistochemistry (20 μm for nodal and paranodal analyses) using a Leica cryostat and processed as floating sections. Cryosections were immersed in PBS blocking solution [0.5% (v/v) Triton X‐100 and 10% fetal calf serum in PBS] for 1 h at ~21°C. Primary antibodies, detailed in Table S1, were diluted in PBS blocking solution and applied to the cryosections overnight at 4°C. Sections were washed thrice in PBS before the application of secondary antibodies conjugated to AlexaFluor‐488, AlexaFluor‐568, AlexaFluor‐647, AlexaFluor‐800 (Invitrogen) [donkey anti‐goat (1:1000), donkey anti‐rabbit (1:1000), donkey anti‐mouse (1:1000), and donkey anti‐rat (1:500), donkey anti‐guinea pig (1:400)], and the nuclear label Hoechst 33342 (1:1000; Invitrogen), diluted in PBS‐blocking solution overnight at 4°C. All sections were again washed in PBS before being mounted using DAKO fluoromount.

Images of the immunohistochemistry were collected using an UltraView Nikon Ti Microscope with Volocity Software (Perkin Elmer), Andor Confocal microscope with Nikon Software (Andor Technology Ltd., Belfast, Northern Ireland), or FluoVIEW slide scanner (Olympus Australia Pty Ltd). Confocal images were collected using standard excitation and emission filters for DAPI, FITC (AlexaFluor‐488), TRITC (AlexaFluor‐568), and CY5 (AlexaFluor‐647), and a 20× air objective lens. For cell density or proportion quantifications, images were collected at 3 μm z‐intervals that spanned an anatomically defined region of interest. Images were stitched together in the *x*–*y* plane to make a single composite image of the CC or MC (separate images of the MC for each brain hemisphere). Cells were identified, and densities or proportions quantified as previously described (Auderset et al. 2020; Cullen et al. 2021).

Imaging and quantification of the node of Ranvier and paranode lengths were also performed as previously described (Cullen et al. 2021). Single *z*‐plane images were collected using a 100× oil immersion lens, and nodes and paranodes were selected for quantification only when a node and its flanking paranodes were intact (flat) within a single *z*‐plane. Length measurements were made using ImageJ (National Institutes of Health), while cell number quantification was undertaken using Adobe Photoshop. Cell densities were calculated in mm^2^, where *x*–*y* measurements were taken from defined regions of interest, and *z*‐plane was not included as all tissue for cell quantification was a set tissue thickness (30 μm). Percentages were used to represent the proportion of differentiated oligodendrocytes (YFP^+^, PDGFRα‐negative) relative to the total number of recombined cells (YFP^+^, OLIG2^+^ cells).

### 2.6 Transmission Electron Microscopy

Mice were terminally anesthetized with a 180 mg/kg i.p. injection of sodium pentobarbitone and transcardially perfused at ~9 mL/min with Karnovsky's fixative (2.5% glutaraldehyde, 2% PFA, 0.25 mM CaCl_2_, 0.5 mM MgCl_2_ in 0.1 M sodium cacodylate buffer). The brains were removed post‐mortem, cut into 1 mm‐thick coronal slices using a brain matrix (Kent Scientific), and post‐fixed in Karnovsky's fixative for 2 h at 21°C. The tissue slices were rinsed and stored in 0.1 M sodium cacodylate buffer at 4°C overnight. The CC was dissected and incubated in 1% osmium tetroxide (OsO_4_)/1.5% potassium ferricyanide [K_3_Fe(III)(CN)_6_] in 0.1 M sodium cacodylate buffer in the dark for 2 h at 4°C, before being dehydrated in ethanol and propylene oxide, and embedded in Epon812 resin. 70 nm ultrathin sections, transecting the CC, were cut using a Leica Ultra‐cut UCT7, and stained with uranyl acetate and lead citrate for imaging on a Hitachi HT7700 transmission electron microscope. G‐ratio, the proportion of myelinated axons, and myelin decompaction were quantified from images collected at 8kV. When axons were ensheathed by myelin with tightly apposed lamellae with clear major dense lines, the axons were classified as having compact myelin. When the myelin structure appeared loose or wavy, with cytoplasmic inclusions, and the major dense lines were no longer clear and concentric, the axon was defined as having uncompacted myelin. Image analysis was carried out using Image J (National Institutes of Health) and involved measuring ≥ 100 axons per mouse.

### 2.7 Motor Behavior

Motor behavior was evaluated as previously described (Cullen et al. 2020). Male and female mice were evaluated prior to tamoxifen administration (P57‐1) to establish baseline performance. Repeat motor testing was performed until P57 + 345. Mice were tested at the same time of day (14:00–17:00), during the light phase of the 12‐h light/dark cycle, and in low‐light conditions (~20 lx). Mice were habituated to the testing room for at least 1 h before commencing.

*Rotarod*: Balance and coordination were assessed using the RotaRod Advanced apparatus (TSE Systems). Mice were trained for 2 weeks at rotation speeds of 10, 20, and 40 rotations per minute (rpm), with an acceleration of 8 rpm/min prior to first data collection to allow for the learning phase of the task. Following training, mice were tested using a RotaRod paradigm (Clark et al. 2018) in which the rotating rod increased speed from 4 to 40 rpm over 300 s, followed by a further 60 s at 40 rpm. For all experiments, “latency to fall” (time required for a mouse to fall off the rotating rod) was recorded. Rotarod performance was assessed three times per session with a 15 min inter‐trial interval, and the average latency was reported.

*Grid walk*: Mice were placed onto a horizontal 2 cm wire mesh grid, positioned 100 cm above the ground, and were left to explore for 5 min. Each mouse was video recorded from underneath the grid using a GOPRO Hero5 camera, and the number of missteps (when the stepping foot slips through the grid) was quantified by an experimenter blind to genotype. We calculated the total number of missteps within the first 100 steps, or over the 5 min if 100 steps were not taken, and this was expressed as a percentage of steps analyzed.

*Beam walk*: Mice were trained to cross a 100 cm length of wooden dowel (10 mm diameter) that was fixed horizontally and positioned 50 cm above the surface of the bench. Individual trials were video recorded at 60 frames/s using a GOPRO Hero5 handheld camera. Foot slips, defined as any paw falling below the level of the beam, were scored from the videos by an experimenter blind to genotype to determine the total number of foot slips made in a single crossing.

*Gait analysis*: Running gait was assessed using the DigiGait Imaging System (Mouse Specifics Inc., MA, USA). Mice were individually placed within the brightly lit plexiglass chamber on the transparent motorized treadmill belt and allowed 5 min to habituate to the chamber. The treadmill was then turned on and moved at 5 cm/s for 5 s before the belt speed increased to 22 cm/s over a 10 s period. A 30 s video of the mouse running at 22 cm/s was recorded at 164 frames/s using a Basler scA640‐74 fc digital camera mounted underneath the treadmill. 3–4 s video segments (~10 steps) were analyzed using DigiGait Analyzer software version 15 (Mouse Specifics Inc., MA, USA). Data for the left and right limbs were pooled, but forelimbs and hindlimbs were analyzed separately.

*Grip strength:* A grip strength meter (Melquest, cat. #GPM‐100) was used to estimate muscular strength as previously described (Cashion et al. 2025). Each mouse was weighed, positioned to grasp the grid mounted on the force gauge, and the equipment set to 0 gF after stabilization. Each mouse was allowed to grasp the grid with all four paws, pulled back over the grid, and the maximum tension recorded. The test was repeated if any of the four paws failed to successfully grasp the grid or the mouse bit or knocked the grid. Five successful trials were recorded per mouse. We calculated force (gF) relative to body weight (g) for each mouse by dividing the mean of five successful tests by body weight.

### 2.8 Dendritic Spine Imaging and Quantification

Mice carrying the *Thy1‐YFPH* transgene underwent transcardial perfusion as described above, but the brain slices were light‐protected during immersion fixation and cryoprotection. Coronal brain cryosections (30 μm) containing the MC were thaw‐mounted directly onto slides and allowed to dry for 15 min before submersion in PBS for 3 min to remove cryomatrix and a brief submersion in dH_2_O to remove salts. Sections were allowed to dry and coverslipped with Prolong Glass Antifade mounting medium (Thermo Fisher Scientific) and high‐precision coverslips (No. 1.5H, Marienfeld Superior). Slides were re‐labeled with a numeric code prior to imaging to ensure the researcher was blind to the experimental conditions.

Dendrite segments were imaged on an Olympus FLUOVIEW FV3000 (BX63LF) laser scanning microscope (514 nm excitation) with a high‐numerical aperture oil‐immersion objective (Olympus Super Apochromat UPLSAPO‐100XO/1.4). Thy1‐YFP^+^ dendrites in pseudo‐randomly selected in the MC. Dendrite segments were selected if they were relatively flat in the *x*–*y* plane and were sufficiently isolated from other YFP^+^ soma and processes to ensure that individual spines could be identified without ambiguity. Apical dendrite segments were identified within layers V and II/III. Basal dendrite segments were only imaged when the soma of origin and the basal dendrite could be clearly identified and captured in the same field of view within layer V. Images were 1024 × 1024 pixels at 0.07–0.1 μm/pixel, collected with a *z*‐step size of 0.09 to 0.2 μm and acquired at 0.2 μs/pixel dwell time and averaged 3× by frame. As the YFP^+^ signal varied, laser power, detector voltage, and offset parameters were adjusted for each image to avoid saturated pixels and minimize background.

Dendritic spine reconstructions were performed in Neurolucida 360 (v.2021.1.3, MBF Bioscience LLC, Williston, VT USA). Traced dendrite segments were at least 10 μm in length, and at least 5 dendrites were reconstructed per mouse. Dendrites were reconstructed with the “user‐guided” manual tree tool then adjusted to accurately fill dendrite volumes. Individual spines were manually selected and the detector sensitivity adjusted for each spine, to ensure that each spine head was accurately reconstructed. Spines also had to meet the following detection parameters: a 10‐voxel minimum size, 0.3 μm minimum height, and a 2.5 μm maximum distance from the dendrite. Spines were semi‐automatically classified into morphological subtypes of thin, mushroom, or stubby, using the Neurolucida 360 multi‐dimensional classifier, with a setting for head‐to‐neck ratio of 1.1, length‐to‐head ratio of 2.2–2.3, and mushroom head size > 0.3 μm. ~15% of spines could not be automatically classified accurately, usually due to patchy fluorescence in the spine neck, and were manually classified based on measured head diameter across the widest portion and head–neck appearance. Dendritic spine densities and proportions of spines classified as each subtype were obtained by batch analysis in Neurolucida Explorer (v.2020.3.1), calculated for each traced dendrite, then averaged per animal (≥ 5 dendrite segments per mouse). Image processing and analysis were performed by a researcher blind to the experimental group.

### 2.9 Electrophysiology

*Extracellular compound action potential* (*CAP*) *recordings:* CAPs were recorded from the adult mouse CC as previously described (Cullen et al. 2021). Briefly, PLP*Myrf* ^*fl/fl*^ and Rα*Myrf* ^*fl/fl*^ mice were killed by cervical dislocation 35–42 days (P57 + 35) or 60–67 days (P57 + 60) after tamoxifen administration, respectively. Brains were rapidly dissected into ice‐cold sucrose solution containing (in mM): sucrose 75, NaCl 87, KCl 2.5, NaH_2_PO_4_ 1.25, NaHCO_3_ 25, MgCl_2_ 7, and CaCl_2_ 0.95. We generated 400 μm acute coronal brain slices, spanning Bregma +0.8 and −0.2, using a Leica VT1200s vibratome. Slices were incubated at ~32°C for 45 min in artificial cerebral spinal fluid (ACSF) containing (in mM): NaCl 119, KCl 1.6, NaH_2_PO_4_ 1, NaHCO_3_ 26.2, MgCl_2_ 1.4, CaCl_2_ 2.4, and glucose 11 (300 ± 5 mOsm/kg), before being transferred to ~21°C ACSF saturated with 95% O_2_/5% CO_2_.

CAPs were evoked by constant current, stimulus‐isolated, square wave pulses (200 ms duration, delivered at 0.2 Hz), using a tungsten bipolar matrix stimulating electrode (FHC; MX21AEW), and were detected using glass recording electrodes (1–3 MΩ) filled with 3 M NaCl. To quantify CAP amplitude, the asymptotic maximum for the short‐latency negative peak (myelinated peak, M) was determined with stimulating and recording electrodes placed 1 mm apart, and by varying the stimulus intensity (0.3–4.0 mA) using an external stimulus isolator (ISO‐STIM 1D), before recording at 80% maximum stimulation. To enhance the signal‐to‐noise ratio, all quantitative electrophysiological analyses were conducted on waveforms that were the average of eight successive sweeps, amplified and filtered (10 kHz low pass bessel) using an Axopatch 200B amplifier (Molecular Devices), and digitized at 100 kHz.

The conduction velocity (CV) of myelinated (M) and unmyelinated (UM) axons was estimated by changing the distance between the stimulating and recording electrodes from 1 to 3 mm of the CC, while holding the stimulus intensity constant (80% maximum). The peak latency of the M and UM axons was measured at each point and graphed relative to the distance separating the electrodes. A linear regression analysis was performed to yield a slope, inverse to the velocity for each brain slice. The average velocity for both CAP components (M, UM) was determined for each mouse, and this value was used for statistical comparison.

*Whole cell patch clamp electrophysiology:* Acute brain slices were generated from PLP*Myrf* ^*fl/fl*^ mice 65–71 days after tamoxifen delivery (P57 + 65), and mini excitatory postsynaptic currents (mEPSCs) recorded from layer V pyramidal neurons in the adult mouse MC, as previously described (Handley et al. 2017). Mice were killed by cervical dislocation, the brain rapidly dissected into ice cold sucrose cutting solution (described above) bubbled with carbogen, and 300 μm coronal brain slices, containing the MC, were cut on a vibratome, transferred to 37°C ACSF (in mM: NaCl 126, KCl 1.6, NaH_2_PO_4_ 1.1, MgCl_2_ 1.4, NaHCO_3_ 26, CaCl_2_ 2.4, glucose 11) for 30 min, and incubated at ~21°C for at least 1 h prior to undertaking recordings. Whole cell patch clamp recordings were performed at ~21°C using a 4–6 MΩ micropipette glass electrode filled with cesium internal solution (in mM: CeMeSO_4_ 125, NaCl 4, KCl 3, MgCl 6H_2_0 1, HEPES 8, EGTA 9, phosphocreatine 10, MgATP 5, Na_2_GTP 1) and an external solution of ACSF with 3 mM TTX (Abcam) and 1 mM picrotoxin. Data were recorded using an Axon Instruments amplifier, sampled at 50 kHz, and high pass filtered at 1 kHz. Layer V pyramidal MC neurons were identified by morphology, had a membrane resistance of 120–150 MΩ, and a membrane capacitance > 70 pF. Cells were excluded if access resistance (Ra) was > 20 MΩ or changed by more than 15% during recordings, or if the response and return to baseline for a hyperpolarizing test pulse (−500 pA) was unstable 3 min after sealing. Data were analyzed using Axograph X (version 1.8.0) to automatically detect mEPSCs within 2.5 standard deviations of a pre‐defined variable‐amplitude template and to measure the amplitude of detected events. Amplitude was measured relative to baseline (mean of 6 ms immediately before each event). Events with negative or zero decay times or decay times above 100 ms were removed post‐processing.

### 2.10 Statistical Analyses

The number of mice analyzed in each group (*n*) or the number of cells, axons, nodes, or paranodes is indicated in the corresponding figure legends. All statistical analyses were performed using Prism 9 (GraphPad Software) or SPSS v.22 (IBM). Data were analyzed by parametric or nonparametric tests as appropriate. Data comparing two groups at a single time point were analyzed using a parametric two‐tailed *t*‐test or a nonparametric Mann–Whitney U (MWU) test. Data distributions were compared using a Kolmogorov–Smirnov (KS) test. Cell counts for lineage tracing, node/paranode length, and proportion of myelinated axons data are presented as mean per mouse ± SD and were analyzed using a two‐way ANOVA with Sidak post‐test multiple comparisons. Behavioral data collected over multiple time points are presented as mean ± SEM and were analyzed using a mixed two‐way ANOVA (sphericity was not assumed), followed by a Sidak‐corrected post‐test. Dendritic spine densities were analyzed by a 3‐way mixed ANOVA that included genotype as a between‐subject factor, and dendrite (apical, basal) and spine morphological type (thin, mushroom, stubby) as repeated measures factors. Significant interactions were followed up with Bonferroni‐corrected simple effects tests comparing genotypes. The average proportion of spines of any morphology is presented as median ± 95% confidence intervals and was analyzed using a Mann–Whitney U test for two‐group comparisons or Kruskal–Wallis test for independent samples (KW) as noted in each figure legend. ANOVA main effects and interactions are reported in the corresponding figure legends. Statistical significance was defined as *p* < 0.05.

## 3 Results

### 3.1 In Adulthood, Conditionally Deleting MyRF From OLs Induces Myelin Loss and Slows Compound Action Potential Conduction

Within mature OLs, the transcription factor MyRF is essential for OL survival and myelin maintenance (Emery et al. 2009; Koenning et al. 2012). To learn how sustaining OLs and myelin influences synaptic plasticity, we conditionally deleted *Myrf* from OLs in young adult mice. Tamoxifen was delivered to P57 control (*Myrf* ^*fl/fl*^) and PLP‐*Myrf* ^*fl/fl*^ (*Plp‐CreERT* ^*::*^ *Myrf* ^*fl/*^ ^*fl*^) mice, and brain tissue was collected 56 days later (P57 + 56). Platelet‐derived growth factor receptor α (PDGFRα)^+^ OPCs and aspartoacylase (ASPA)^+^ mature OLs were readily detected in the MC and CC of control and PLP*Myrf* ^*fl/fl*^ mice (Figure 1A–D), and the small but significant increase in OPC density in the CC of PLP*Myrf* ^*fl/fl*^ mice (Figure 1E) was considered indicative of OPCs responding to an induced white matter injury (Koenning et al. 2012; Watanabe et al. 2002). While ASPA^+^ OL density appeared normal in the MC and CC of P57 + 56 PLP*Myrf* ^*fl/fl*^ mice (Figure 1F), this population would include a mixture of surviving and dying OLs and newborn OLs (Duncan et al. 2024; Koenning et al. 2012). Demyelination and OL death would be ongoing at this timepoint, as they have been reported to peak ~10–12 weeks after tamoxifen delivery to PLP*Myrf* ^*fl/fl*^ mice and overlap with remyelination, which commences ~8 weeks after tamoxifen delivery (Duncan et al. 2024; Hartley et al. 2019; Koenning et al. 2012) ‐ the end point of our study. To determine whether the deletion of *Myrf* from OLs prevented myelin maintenance and induced demyelination, we next stained brain cryosections to quantify myelin levels and gliosis (Figure 1G–I). Myelin basic protein (MBP) labelling was reduced (Figure 1I), and a greater area of the CC was covered by ionized calcium‐binding adapter protein 1 (Iba1)^+^ (Figure 1J) and glial fibrillary acidic protein (GFAP)^+^ (Figure 1K) pixels. The marked MBP loss and accompanying gliosis indicated that myelin was compromised in PLP*Myrf* ^*fl/fl*^ mice by P57 + 56.

**FIGURE 1:**
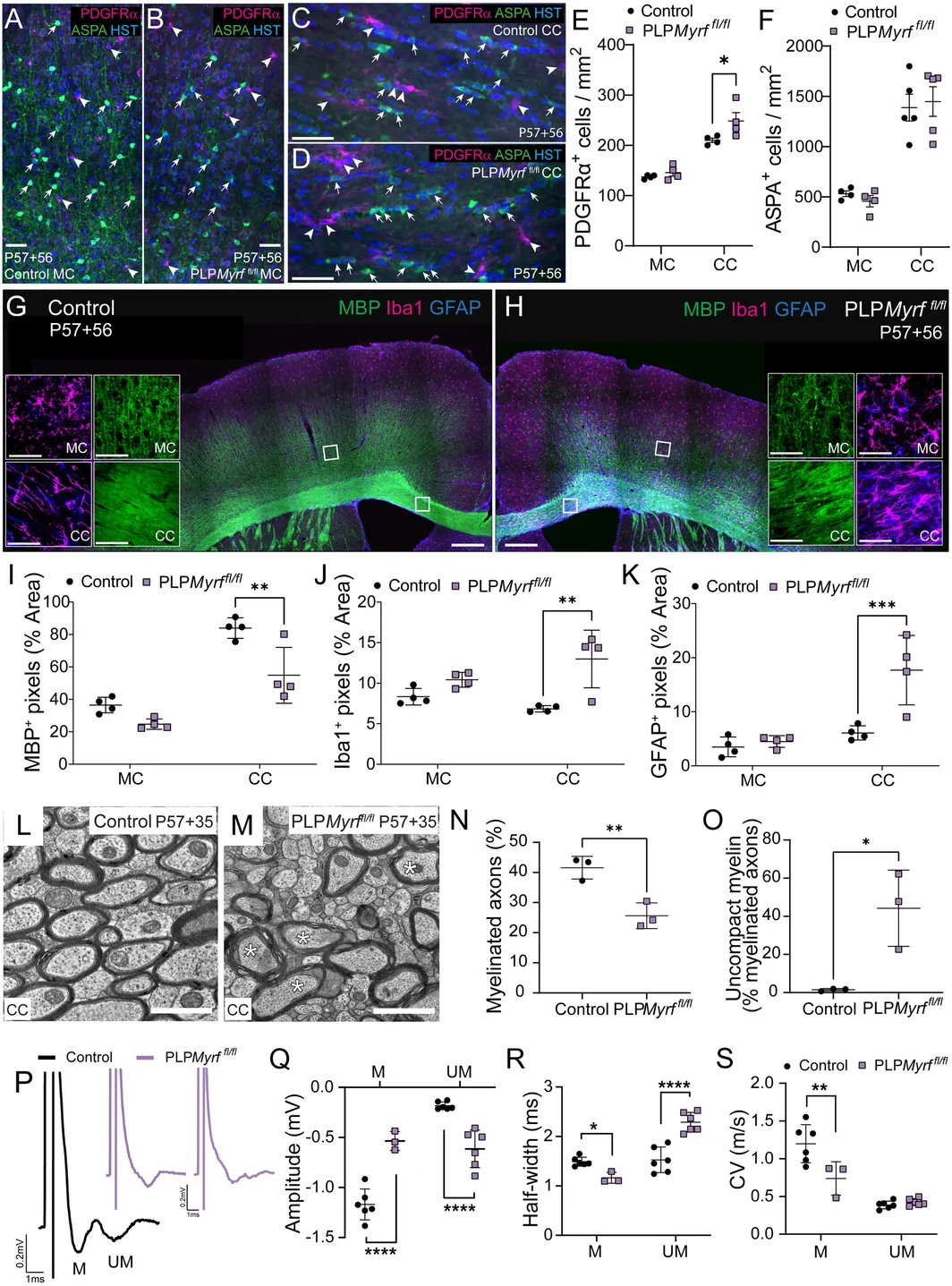
Conditionally deleting Myrf from OLs at P57 results in myelin loss and impaired action potential conduction. (A–D) PDGFRα (magenta) and ASPA (green) labelling in the motor cortex (MC) and corpus callosum (CC) of control (A, C) and PLP*Myrf* ^*fl/fl*^ (B, D) mice at P57 + 56. Scale bar represents 30 μm (A, B) and 40 μm (C, D). (E) Quantification of PDGFRα^+^ OPC density in the MC and CC of control (black circles; *n* = 4) and PLP*Myrf* ^*fl/fl*^ (purple squares; *n* = 4) mice at P57 + 56. [Two‐way ANOVA: Interaction F(1,12) = 2.59, *p* = 0.133; region F(1,12) = 89.43, *p* = <0.0001; genotype F(1,12) = 6.44, *p* = 0.026]. (F) Quantification of ASPA^+^ OL density in the MC (*n* = 4 per group) and CC (*n* = 5 per group) of control and PLP*Myrf* ^*fl/fl*^ mice at P57 + 56. [Two‐way ANOVA: Interaction F(1,14) = 0.34, *p* = 0.568; region F(1,14) = 63.25, *p* = <0.0001; genotype F(1,14) = 0.004, *p* = 0.951]. (G, H) MBP (green), Iba1 (magenta), and GFAP labeling (blue) in the MC and CC of control (G) and PLP*Myrf* ^*fl/fl*^ (H) mice at P57 + 56. Scale bar represents 200 and 40 μm (inset). (I) Quantification of the proportion of area containing MBP^+^ pixels in the MC and CC of control (*n* = 4) and PLP*Myrf* ^*fl/fl*^ (*n* = 4) mice at P57 + 56. [Two‐way ANOVA: Interaction F(1,12) = 3.26, *p* = 0.096; region F(1,12) = 65.16, *p* = <0.0001; genotype F(1,12) = 18.04, *p* = 0.0011]. (J) Quantification of the proportion of area containing Iba1+ pixels in the MC and CC of control (*n* = 4) and PLP*Myrf* ^*fl/fl*^ (*n* = 4) mice at P57 + 56. [Two‐way ANOVA: Interaction F(1,12) = 4.49, *p* = 0.055; region F(1,12) = 0.31, *p* = 0.58; genotype F(1,12) = 18.48, *p* = 0.0010]. (K) Quantification of the proportion of area containing GFAP+ pixels in the MC and CC of control (*n* = 4) and PLP*Myrf* ^*fl/fl*^ (*n* = 4) mice at P57 + 56. [Two‐way ANOVA: Interaction F(1,12) = 9.52, *p* = 0.009; region F(1,12) = 21.08, *p* = 0.0006; genotype F(1,12) = 13.43, *p* = 0.0032]. (L, M) Transmission electron micrographs from the CC of P57 + 35 control and PLP*Myrf* ^*fl/fl*^ mice. Scale bar represents 1µm. Large white asterisks denote axons with uncompacted myelin. (N) Quantification of the proportion of myelinated axons in the CC of control (*n* = 3) and PLP*Myrf* ^*fl/fl*^ (*n* = 3) mice at P57 + 35. [Unpaired *t*‐test: *T* = 4.85, df = 4; *p* = 0.0083]. (O) Quantification of the proportion of myelinated callosal axons with loose, uncompact myelin in control (*n* = 3) and PLP*Myrf* ^*fl/fl*^ (*n* = 3) mice at P57 + 35. [Unpaired *t*‐test: *T* = 3.703, df = 4; *p* = 0.0208]. (P) Example CAP traces from the CC of P57 + 35 control (black) and PLP*Myrf* ^*fl/fl*^ (purple) mice. Please note that the top of the stimulus artifact has been cropped from the traces to allow clearer visualization of the smaller compound action potential peaks. (Q) Amplitude (mV) of the myelinated (M) and unmyelinated (UM) peaks of callosal CAPs measured in P57 + 35 control (*n* = 6) and PLP*Myrf* ^*fl/fl*^ (*n* = 6) mice. [Two‐way ANOVA: Interaction F(1,17) = 71.70, *p* < 0.0001; peak F(1,17) = 52.19, *p* < 0.0001; genotype F(1,17) = 2.61, *p* = 0.124]. (R) Half‐width (ms) of the myelinated (M) and unmyelinated (UM) peaks of callosal CAPs measured in P57 + 35 control (*n* = 6) and PLP*Myrf* ^*fl/fl*^ (*n* = 6) mice. [Two‐way ANOVA: Interaction F(1,17) = 40.36, *p* < 0.0001; peak F(1,17) = 55.89, *p* < 0.0001; genotype F(1,17) = 6.42, *p* = 0.021]. (S) Conduction velocity for myelinated (M) and unmyelinated (UM) axons measured from callosal CAP traces recorded from P57 + 35 control (*n* = 6) and PLP*Myrf* ^*fl/fl*^ (*n* = 6) mice. [Two‐way ANOVA: Interaction F(1,17) = 11.48, *p* = 0.0035; peak F(1,17) = 58.48, *p* < 0.0001; genotype F(1,17) = 8.28, *p* = 0.01]. Graphs show mean ± SD. *p < 0.05; **p < 0.01; ***p < 0.001; ****p < 0.0001 by Bonferroni's post‐test or *t*‐test (N, O).

To establish myelin loss as an early consequence of *Myrf*‐deletion we performed immunohistochemistry and transmission electron microscopy (TEM). We found that MBP labelling was already significantly reduced in the CC of P57 + 28 PLP*Myrf* ^*fl/fl*^ mice (Figure S2). Furthermore, significantly fewer CC axons were myelinated in P57 + 35 PLP*Myrf* ^*fl/fl*^ mice compared to controls (Figure 1L–N), and a higher proportion of myelinated axons showed evidence of myelin decompaction (Figure 1O). By analysing the axons that retained compact myelin, we found that the myelinatedaxong‐ratio was equivalent in P57 + 35 PLP*Myrf* ^*fl/*^ ^*fl*^ and control mice (Figure S2). While the average diameter of unmyelinated and myelinated CC axons remained unchanged by genotype, the distribution of unmyelinated and myelin axon diameters was shifted towards larger axons in P57 + 30 PLP*Myrf* ^*fl/fl*^ mice (Figure S2), suggesting a small degree of axon swelling was induced in the demyelinating CC. When we measured CAP conduction across the CC in acute brain slices from P57 + 35 control mice, we consistently observed two peaks (Figure 1P) that corresponded to action potentials conduction by the myelinated (M) and unmyelinated (UM) axon populations (Cullen et al. 2021). By contrast, CAP recordings from PLP*Myrf* ^*fl/fl*^ brain slices had a smaller or absent M axon peak, but a significantly larger UM axon peak (Figure 1P), consistent with a large proportion of previously myelinated CC axons transitioning to a functionally demyelinated state. When PLP*Myrf* ^*fl/fl*^ mice retained functionally myelinated axons, that is, they had a M axon peak, the amplitude and half‐width of the peak (Figure 1Q,R), and the calculated CAP conduction velocity were significantly reduced (Figure 1S), suggesting that even the remaining myelinated axons were functionally compromised. In all PLP*Myrf* ^*fl/fl*^ mice, the amplitude and half‐width of the UM axon peak were significantly increased (Figure 1Q,R), indicating that the large number of demyelinated CC axons that increased the amplitude of the UM peak, had more heterogenous conduction velocities than UM axons in control mice. As our TEM and CAP data indicate that PLP*Myrf* ^*fl/fl*^ mice experience severe functional demyelination by P57 + 35, and our immunohistochemical data indicate that demyelination is sustained from P57 + 28 until P57 + 56, it is likely that behavioral modalities, including motor coordination and performance are also impaired over this time course (Koenning et al. 2012).

### 3.2 The Adult Deletion of Myrf From OLs Leads to Motor Dysfunction

To determine whether motor function is impaired in PLP*Myrf* ^*fl/fl*^ mice, motor performance was evaluated 1 day prior to tamoxifen delivery and then weekly for 6–8 weeks (up to P57 + 56). Mice were placed on the rotarod to detect large changes in motor circuit and/or muscle function, such as those seen in preclinical models of amyotrophic lateral sclerosis (Clark et al. 2018). The rotarod performance of PLP*Myrf* ^*fl/fl*^ mice appeared poor relative to controls, but this did not reach statistical significance at any individual timepoint (Figure 2A). By contrast, when mice were placed on the grid‐walk to evaluate dexterous fine motor skill, we found that the performance of PLP*Myrf* ^*fl/fl*^ mice was significantly impaired by P57 + 35 (Figure 2B). Similarly, control mice made few beam‐walk foot‐slip errors at any age, but the performance of PLP*Myrf* ^*fl/fl*^ mice had declined significantly by P57 + 21 (Figure 2C).

**FIGURE 2:**
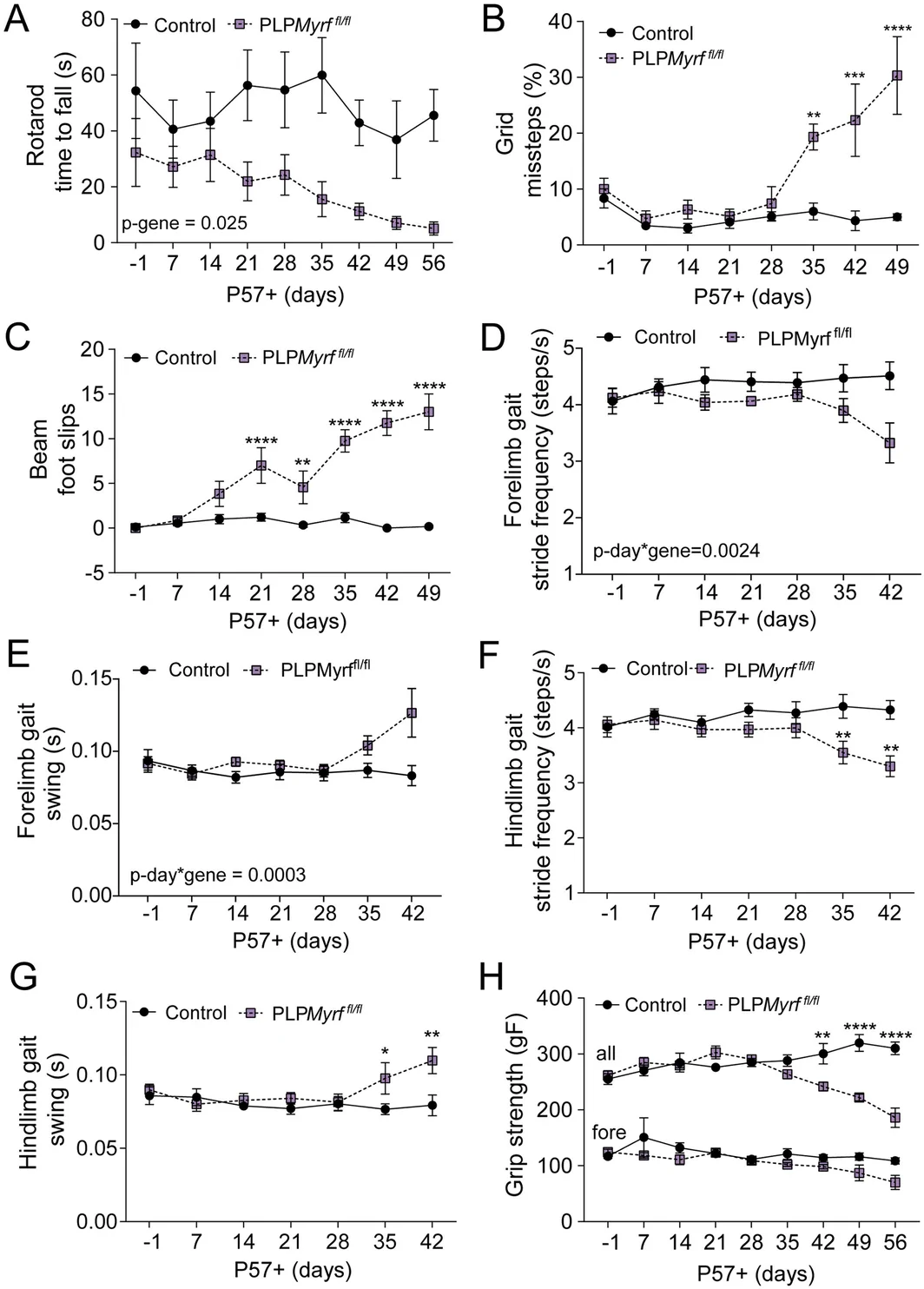
The conditional deletion of Myrf from OLs at P57 impairs motor function within 21 days. (A) Time taken for control (black circles) and PLP*Myrf* ^*fl/fl*^ (purple squares) mice to fall from the rotarod at P57 − 1 to P57 + 56. [Restricted Maximum Likelihood (REML) mixed effects model: Interaction F(8,84) = 0.801 *p* = 0.603; day F(2.86, 30.04) = 1.73 *p* = 0.18; genotype F(1,14) = 6.31 *p* = 0.025]. (B) Proportion of missteps made by control and PLP*Myrf* ^*fl/fl*^ mice performing the grid walk test at P57 − 1 to P57 + 49. [Restricted Maximum Likelihood (REML) mixed effects model: Interaction F(7,64) = 7.601 *p* < 0.0001; day F(2.52, 23.05) = 8.626 *p* = 0.0008; genotype F(1,14) = 27.26 *p* < 0.0001]. (C) Number of foot slips made by control and PLP*Myrf* ^*fl/fl*^ mice performing the beam walk test at P57 − 1 to P57 + 49. [Restricted Maximum Likelihood (REML) mixed effects model: Interaction F(7,74) = 14.2 *p* < 0.0001; day F(1.7, 18.2) = 14.65 *p* = 0.0003; genotype F(1,14) = 91.73 *p* < 0.0001]. (D, E) Average forelimb stride frequency (D) and limb swing time (E) of control and PLP*Myrf* ^*fl/fl*^ mice during treadmill (DigiGait) running from P57 − 1 to P57 + 42. [Restricted Maximum Likelihood (REML) mixed effects model: *Stride frequency* (Interaction F(6,52) = 4.41 *p* = 0.0011; day F(2.99, 25.94) = 1.53 *p* = 0.229; genotype F(1,12) = 3.81 *p* = 0.074); *Swing* (Interaction F(6,53) = 5.24 *p* = 0.0003; day F(1.930, 17.05) = 2.47 *p* = 0.116; genotype F(1,13) = 3.145 *p* = 0.099)]. (F, G) Average hindlimb stride frequency (F) and limb swing time (G) of control and PLP*Myrf* ^*fl/fl*^ mice during treadmill (DigiGait) running from P57 − 1 to P57 + 42. [Restricted Maximum Likelihood (REML) mixed effects model: *Stride frequency* (Interaction F(6,52) = 4.41 *p* = 0.0011; day F(2.99, 25.94) = 1.53 *p* = 0.229; genotype F(1,12) = 3.81 *p* = 0.074); *Swing* (Interaction F(6,52) = 5.42 *p* = 0.0002; day F(2.02, 17.48) = 3.06 *p* = 0.072; genotype F(1,12) = 1.68 *p* = 0.21)]. (H) Grip strength for all paws and forepaws only of control and PLP*Myrf* ^*fl/fl*^ mice from P57 − 1 to P57 + 56. [Restricted Maximum Likelihood (REML) mixed effects model: *All paws* (Interaction F(8,84) = 14.27 *p* < 0.0001; day F(3.16, 33.25) = 4.37 *p* = 0.0096; genotype F(1,14) = 10.02 *p* = 0.0069); *Fore paws* (Interaction F(8,84) = 0.79 *p* = 0.62; day F(1.57, 16.50) = 2.08 *p* = 0.16; genotype F(1,14) = 4.18 *p* = 0.06)]. Graphs show mean ± SEM. Control *n* = 6–9; PLPMyrf^*fl/fl*^ *n* = 4–7. *p < 0.05; **p < 0.01; ***p < 0.001; ****p < 0.0001 by Bonferroni's post‐test or *t*‐test. See also Figure S1.

We predicted that preventing OL and myelin maintenance could also disrupt established motor patterns, like those that underpin gait, and found that gait was altered by P57 + 35 (Figures 2D–G and S1). Forelimb gait parameters were largely unaffected over the testing period (Figures 2D,E and S1), however, PLP*Myrf* ^*fl/fl*^ hindlimb stride frequency was reduced (Figure 2F) and gait swing time was increased (Figure 2G) by P57 + 35. These data are consistent with previous reports of ataxia following the deletion of *Myrf* from OLs (Duncan et al. 2024; Koenning et al. 2012) and suggest that the relative movement of fore and hindlimbs becomes uncoordinated. The majority of PLP*Myrf* ^*fl/fl*^ mice (~70%) failed to walk on the Digigait treadmill after P57 + 42, preventing further reliable recordings from being obtained. As PLP*Myrf* ^*fl/fl*^ forepaw grip strength was normal, but the grip strength of all four paws was significantly weaker from P57 + 42 (Figure 2H), myelin may be more rapidly compromised in circuits that regulate hind paw movement or the ability of mice to coordinate movement of the fore and hind paws.

### 3.3 OL and Myelin Maintenance Allow Layer V Pyramidal Neurons to Retain a High Proportion of Thin, Highly Plastic, Dendritic Spines

In the MC, layer V pyramidal neurons have small spines protruding from their apical and basal dendrites that correspond to the postsynaptic compartments for inputs from excitatory cortical and thalamic neurons (Geng et al. 2022). To determine whether the myelin laid down before 2 months of age regulates dendritic spine density or structural plasticity in adulthood, we crossed PLP*Myrf* ^*fl/fl*^ and *Thy1‐YFPH* transgenic mice. The *Thy1‐YFPH* transgene drives YFP expression in a subset of MC pyramidal neurons, supporting visualization of the apical and basal dendrites and dendritic spines (Figure 3A,B). Individual YFP^+^ pyramidal neurons were identified in layer V of the MC of P57 + 60 *Thy1‐YFPH* control and PLP*Myrf* ^*fl/fl*^ mice, and high‐resolution confocal images were collected along the apical and basal dendrites for 3D reconstruction (Figure 3C,D). The overall density of dendritic spines was ~19 spines/10 μm length of apical or basal dendrite and was equivalent in control and PLP*Myrf* ^*fl/fl*^ mice (Figure 3E). As spines undergo structural changes that reflect their functional state, we next classified each spine into one of three morphological types: thin, stubby, or mushroom (Figure 3B–D). The apical dendrites of PLP*Myrf* ^*fl/fl*^ mice had an increased density of stable mushroom spines compared to control mice (Figure 3F). The density of mushroom spines was similarly increased along the basal dendrites of PLP*Myrf* ^*fl/fl*^ mice, at the expense of thin, plastic spines (Figure 3F).

**FIGURE 3:**
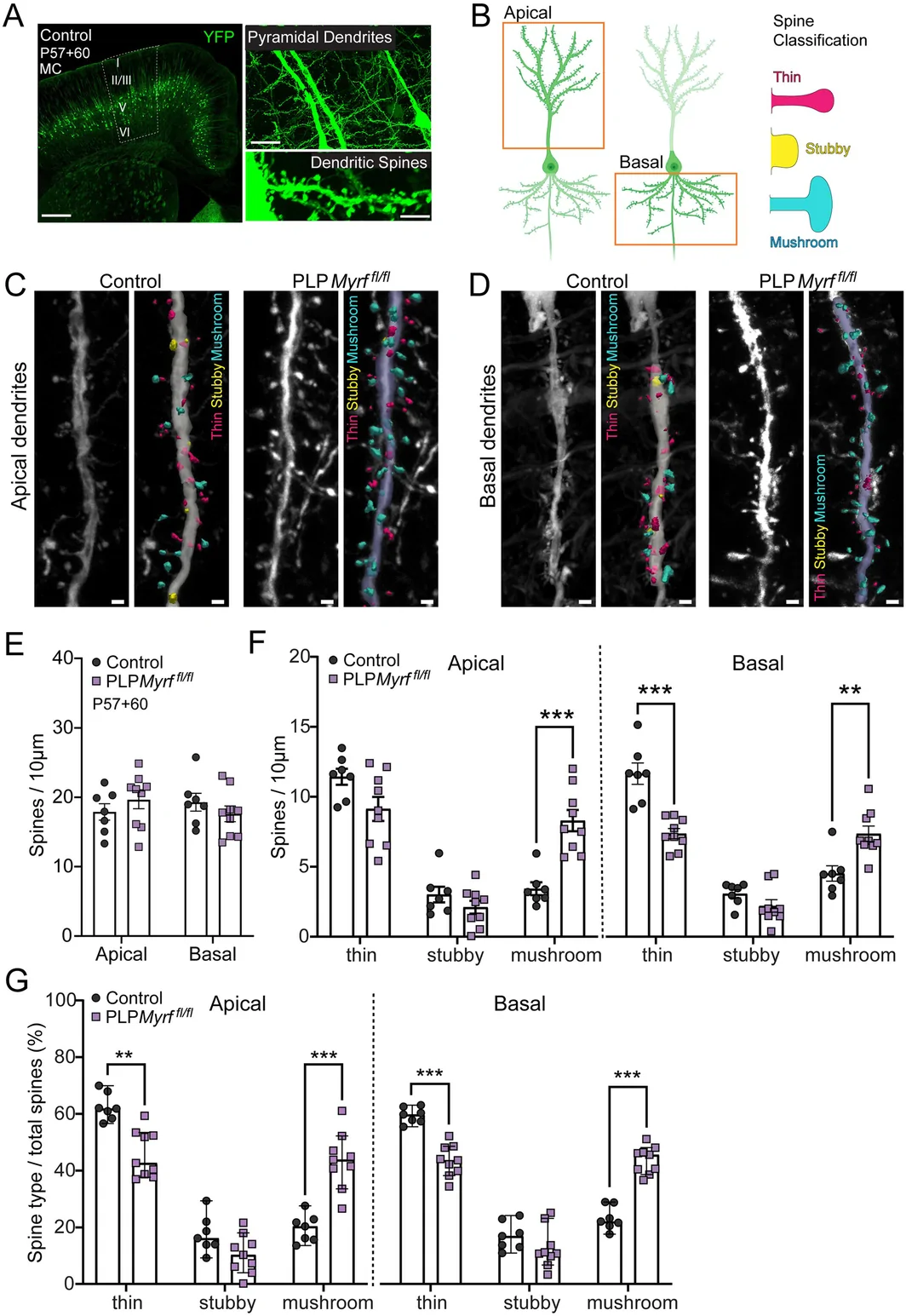
At P57, the conditional deletion of Myrf from OLs reduces the proportion of thin “learning” spines on layer V pyramidal neuron dendrites. (A) YFP (green) labelling of layer V pyramidal neurons in the MC, including dendrites and dendritic spines, in P57 + 60 *Thy1‐YFPH* mice. Scale bars represent 200 μm (left), 40 μm (top right), and 5 μm (bottom right). (B) Schematic representation of apical and basal dendrites of cortical layer V pyramidal neurons and the morphology of thin (magenta), stubby (yellow), and mushroom (cyan) spine type classifications. (C, D) Representative images and rendered traced apical (A) and basal (B) dendrite segments of Thy1‐YFP^+^ layer V pyramidal neuron in P57 + 60 control and PLP*Myrf* ^*fl/fl*^ mice. Rendered images show example spines classified by morphology as thin (magenta), stubby (yellow), or mushroom (cyan) subtypes. Scale bars represent 1 μm. (E) Mean dendritic spine density (number of spines per 10 μm dendrite length) for apical and basal dendrites. Overall spine density did not differ significantly between genotypes in apical or basal dendrites [two‐way mixed ANOVA; genotype × dendrite interaction: F(1,14) = 2.533, *p* = 0.134; genotype (control, PLP*Myrf* ^*fl/fl*^): F(1,14) = 0.104, *p* = 0.751; dendrite (within‐subjects: Apical, basal): F(1,14) = 0.237, *p* = 0.634]. (F) Mean spine densities (number of spines per 10 μm dendrite length) of each morphological subtype (thin, mushroom, stubby) for apical and basal dendrites. Bars = means. Error bars represent SEM. Densities of subtypes were significantly different between control and PLP*Myrf* ^*fl/fl*^ mice [3‐way mixed ANOVA run with within‐subject factors: Dendrite (apical, basal), spine type (thin, mushroom, stubby) and between‐subjects factor: Genotype (control, PLP*Myrf* ^*fl/fl*^); 3‐way interaction: Dendrite*spine type* genotype, F(2,28) = 1.327, *p* = 0.281); two‐way interactions: Spine type* genotype: F(2,28) = 39.56, *p* < 0.001; dendrite*genotype: F(1,14) = 2.516, *p* = 0.135; dendrite*spine type: F(2,28) = 0.915, *p* = 0.412; dendrite: F(1,14) = 0.249, *p* = 0.625; spine type: F(2,28) = 159.973, *p* < 0.001; genotype: F(1,14) = 0.09, 0.765]. (G) The proportion of dendritic spines of each morphological subtype (spine type/total spines × 100). Bars = median. Error bars represent 95% CIs. Control versus PLP*Myrf* ^*fl/fl*^ mean ranks Mann–Whitney U, Bonferroni‐corrected *p*‐values; apical dendrites percent thin: *Z* = −3.123, *p* = 0.002; apical dendrites percent mushroom: *Z* = 3.228, *p* = 0.001. Basal dendrites percent thin: *Z* = −3.334, *p* < 0.001; Basal dendrites percent mushroom: *Z* = 3.334, *p* < 0.001. Percentage of spines classified as stubby spines did not differ between genotypes in apical or basal dendrites (*p*‐values > 0.05). Data points represent individual mice (mean of ≥ 5 dendrite segments per mouse). *p < 0.05, **p < 0.01, ***p < 0.001 represent Bonferroni‐adjusted *p*‐values.

The maturation of thin spines into mushroom spines is a structural correlate of long‐term potentiation, with larger spine heads having a higher concentration of AMPA receptors (Matsuzaki et al. 2004). As a predominance of thin spines was replaced by an approximately equal proportion of thin and mushroom spines in PLP*Myrf* ^*fl/fl*^ mice (Figure 3G), we predicted that the layer V MC pyramidal neurons would generate larger mEPSCs in response to spontaneous quantal glutamate release. To evaluate this, we generated acute coronal brain slices from P57 + 65 control or PLP*Myrf* ^*fl/fl*^ mice for whole cell patch clamp electrophysiology. The capacitance of layer V pyramidal neurons was equivalent in control and PLP*Myrf* ^*fl/fl*^ mice, and the membrane resistance was also unaffected by genotype (Figure 4A). When mEPSCs were recorded at the soma (Figure 4B), the average amplitude of mEPSC events was significantly increased in PLP*Myrf* ^*fl/fl*^ mice (Figure 4C). This was associated with a rightward shift in the cumulative distribution plot of mEPSC amplitude (Figure 4D). The released glutamate vesicles produced larger postsynaptic depolarizations, which is consistent with more spines being mushroom spines (Figure 3G). mEPSC frequency was also elevated in PLP*Myrf* ^*fl/fl*^ mice (Figure 4E), which could reflect an increase in the number of presynaptic sites or an increased probability of presynaptic vesicle release in PLP*Myrf* ^*fl/fl*^ mice. These data indicate that previously laid down myelin ensures that neurons retain a high proportion of thin plastic spines by limiting the number of synapses that become stable mushroom spines. In this way, myelin also regulates glutamate sensitivity in the adult mouse MC.

**FIGURE 4:**
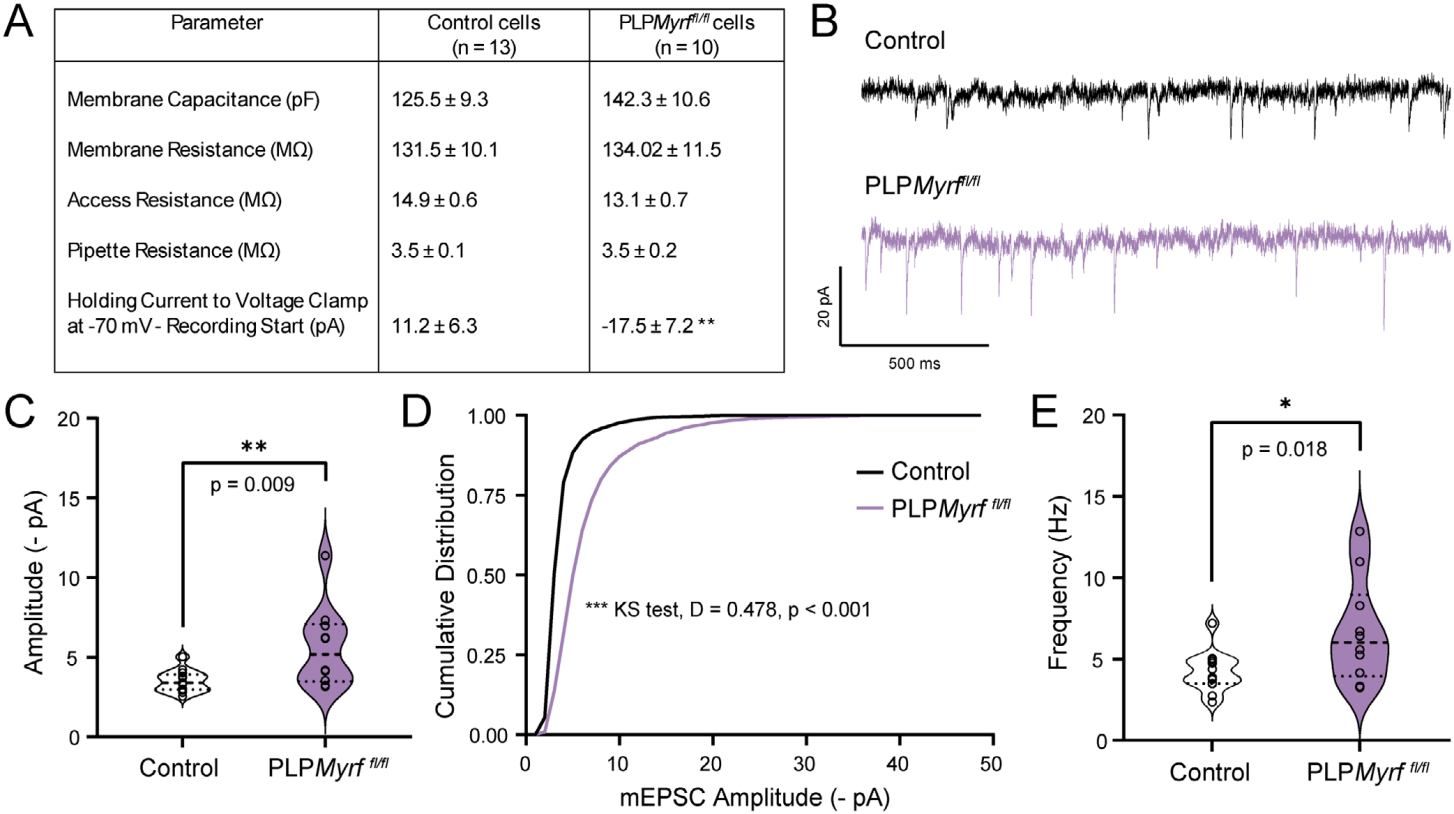
Layer V pyramidal MC neurons generate larger mEPSCs in response to vesicular glutamate release in P57 + 65 PLPMyrf^*fl/fl*^ mice. (A) Table of membrane properties and other measurements acquired during the whole cell patch clamp recordings of layer V pyramidal neurons from P57 + 65 control and PLP*Myrf* ^*fl/fl*^ mice. Parameters were compared between groups using unpaired two‐tailed (independent samples) *t*‐tests. (B) Representative trace showing mEPSCs recorded from layer V pyramidal neurons of P57 + 65 control or PLP*Myrf* ^*fl/fl*^ mice. (C) Average amplitude of mEPSCs recorded from layer V pyramidal neurons of P57 + 65 control and PLP*Myrf* ^*fl/fl*^ mice. Recordings were taken from *n* = 13 neurons from slices of *n* = 5 P57 + 65 control mice, and *n* = 10 neurons from slices of *n* = 3 PLP*Myrf* ^*fl/fl*^ mice. Average amplitude was compared by two‐tailed unpaired *t*‐test (*t* = 2.892), *p* < 0.01. (D) Cumulative distribution plots of amplitudes for mEPSCs recorded from layer V pyramidal neurons in P57 + 65 control and PLP*Myrf* ^*fl/fl*^ mice. The distributions were compared by a two‐sample Kolmogorov–Smirnov test: D = 0.478, *p* < 0.001. (E) Average frequency of mEPSCs recorded from the soma of layer V pyramidal neurons of P57 + 65 control and PLP*Myrf* ^*fl/fl*^ mice. Recordings were taken from *n* = 13 neurons from 5 individual P57 + 65 control mice, and *n* = 10 neurons from 3 individual PLP*Myrf* ^*fl/fl*^ mice. Average frequency was compared by two‐tailed unpaired *t*‐test (*t* = −2.572), *p* < 0.05. Data points in C and E represent individual neurons. *p < 0.05, **p < 0.01, ***p < 0.001.

### 3.4 The Conditional Deletion of Myrf From Adult OPCs Prevents Further OL Addition and Myelination

OLs are added to the brain throughout adulthood (Bacmeister et al. 2020; Rivers et al. 2008) and, along with synaptic plasticity (Bailey et al. 2015), are required for memory consolidation (Pan et al. 2020; Steadman et al. 2020). To evaluate the capacity of adult‐born OLs to influence synapse number or structure, even under basal conditions, we conditionally deleted *Myrf* from OPCs in the adult mouse CNS and prevented new OL maturation. Tamoxifen was administered to P57 control (*Pdgfrα‐CreER:: Rosa26‐YFP*) and Rα*Myrf* ^*fl/fl*^ (*Pdgfrα‐CreER* ^*::*^ *Rosa26‐YFP:: Myrf* ^*fl/fl*^) transgenic mice. As new OPCs can be generated from neural stem cells in the adult mouse subventricular zone (Xing et al. 2014; Young et al. 2010), tamoxifen was also administered once every 90 days throughout the study to capture these cells. Immunohistochemistry was performed on coronal brain cryosections to detect PDGFRα, OLIG2, and YFP in the MC and CC of control and Rα*Myrf* ^*fl/fl*^ mice, from P57 + 30 to P57 + 345 (Figure 5A–L). The density of PDGFRα^+^ OPCs was consistent over time in the MC and CC, equivalent in control and Rα*Myrf* ^*fl/fl*^ mice, and the proportion of OPCs that were recombined was unaffected by genotype (Figure S3). In the MC, control mice had significantly more YFP^+^ newborn OLs (PDGFRα‐neg OLIG2^+^ YFP^+^) than Rα*Myrf* ^*fl/fl*^ mice by P57 + 30 (Figure 5M). In the CC of P57 + 30 Rα*Myrf* ^*fl/fl*^ mice, the proportion of YFP^+^ cells that were PDGFRα‐neg OLIG2^+^ newborn OLs was reduced by ~59% (Figure 5N). By P57 + 120, new OL number was reduced by ~79% in the MC (Figure 5M) and ~82% in the CC (Figure 5N). The reduction in newborn OLs was largely due to the impaired survival of premyelinating OLs, as P57 + 120 Rα*Myrf* ^*fl/fl*^ mice had ~76% fewer YFP^+^ BCAS1^+^ (breast carcinoma amplified sequence 1) premyelinating OLs in the MC and ~84% fewer in the CC than control mice (Figure 5O–T). We also noted that the proportion of YFP^+^ cells that were OLs declined significantly in the CC of control mice between P52 + 120 and P57 + 345 (Figure 5N). OL die in older mice (Hill et al. 2018), including within white matter tracts (Zhi et al. 2023), and our data may suggest that OLs added to the CC in adulthood are highly susceptible to age‐related loss.

**FIGURE 5:**
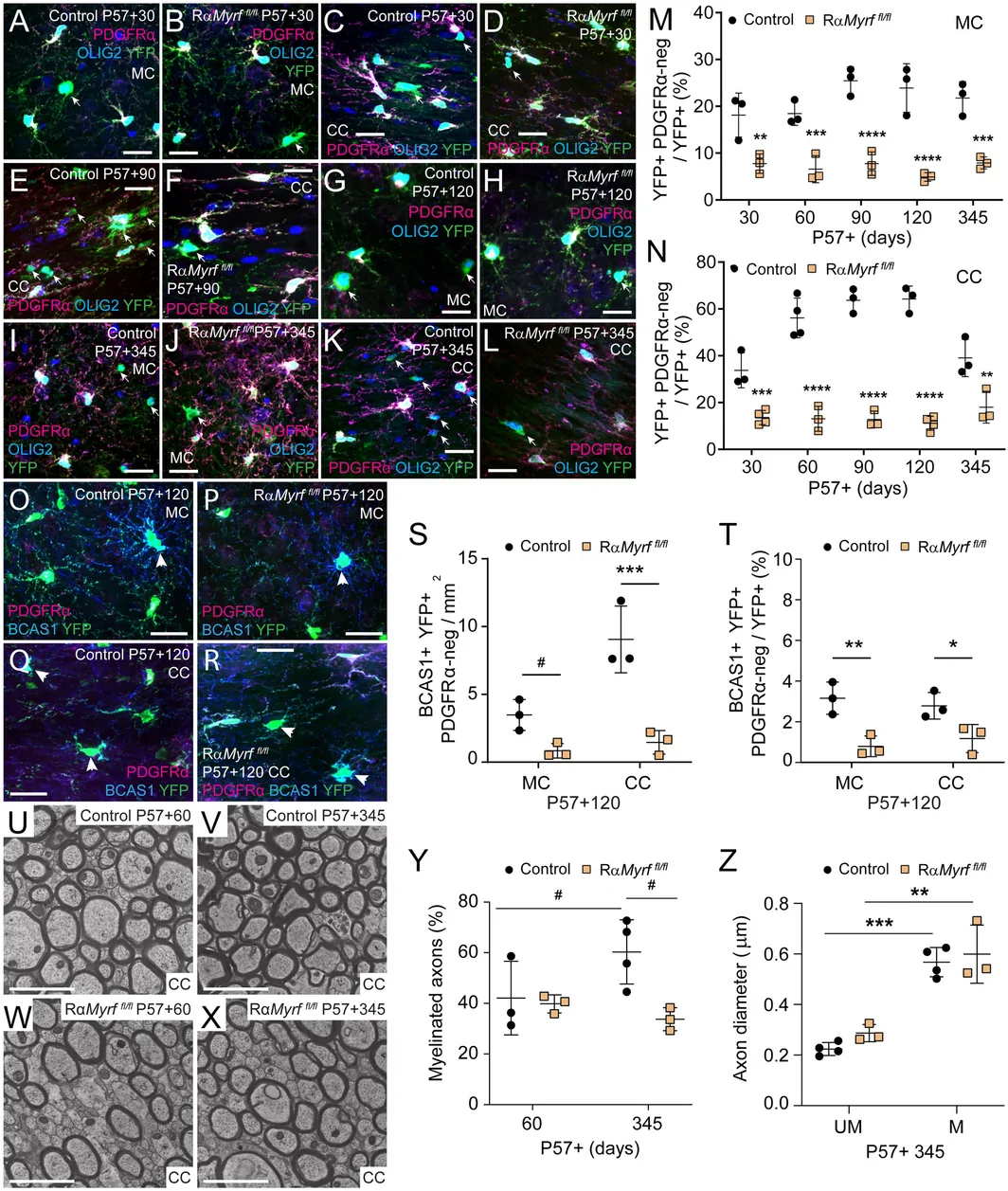
Conditionally deleting Myrf from OPCs at P57 blocks adult oligodendrogenesis and myelination. (A–L) Confocal images from the motor cortex (MC) and corpus callosum (CC) of control and Rα*Myrf* ^*fl/fl*^ mice, stained to detect OPCs (PDGFRα, magenta), cells of the OL lineage (OLIG2, blue), and YFP (green). (M) Quantification of the proportion of YFP^+^ cells that are PDGFRα‐negative OLIG2^+^ OLs in the MC of control and Rα*Myrf* ^*fl/fl*^ mice between P57 + 30 and P57 + 345 [Two‐way between‐subjects ANOVA, timepoint × genotype interaction: F(4,20) = 2.100, *p* = 0.1187; timepoint: F(4,20) = 1.627, *p* = 0.2066; genotype: F(1,20) = 163.0, *p* < 0.001]. (N) Quantification of the proportion of YFP^+^ cells that are PDGFRα‐negative OLIG2^+^ OLs in the CC of control and Rα*Myrf* ^*fl/fl*^ mice between P57 + 30 and P57 + 345 [Two‐way between‐subjects ANOVA, timepoint × genotype interaction: F(4,23) = 11.91, *p* < 0.001; timepoint: F(4,23) = 7.459, *p* < 0.001; genotype: F(1,23) = 330.0, *p* < 0.001]. (O–R) Confocal images from the CC and MC of P57 + 120 control and Rα*Myrf* ^*fl/fl*^ mice, stained to detect OPCs (PDGFRα, magenta), premyelinating OLs (BCAS1, blue), and YFP (green). (S) Density of BCAS1^+^ YFP^+^ premyelinating OLs (PDGFRα‐neg) in the MC and CC of control (black circles) and Rα*Myrf* ^*fl/fl*^ (orange squares) mice at P57 + 120. Two‐way ANOVA: Interaction F(1,8) = 8.75 *p* = 0.018; region F(1,8) = 13.67 *p* = 0.0061; genotype F(1,8) = 37.38 *p* = 0.0003. (T) Proportion of total YFP^+^ cells that were BCAS1^+^ premyelinating OLs (PDGFRα‐neg) in the MC and CC of control (black circles) and Rα*Myrf* ^*fl/fl*^ (orange squares) mice at P57 + 120. Two‐way ANOVA: Interaction F(1,8) = 0.96 *p* = 0.35; region F(1,8) = 0.0003 *p* = 0.98; genotype F(1,8) = 26.50 *p* = 0.0009. (U–X) Transmission electron micrographs of sagittal sections transecting the CC of P57 + 60 and P57 + 345 control and Rα*Myrf* ^*fl/fl*^ mice. Scale bars represent 2 μm. (Y) Proportion of callosal axons that are myelinated in the CC of control and Rα*Myrf* ^*fl/fl*^ mice at P57 + 60 and P57 + 345. [Two‐way ANOVA, timepoint × genotype interaction: F(1,9) = 4.356, *p* = 0.0665; timepoint: F(1,9) = 1.091, *p* = 0.3235; genotype: F(1,9) = 6.158, *p* = 0.0349]. (Z) The average diameter of unmyelinated (UM) and myelinated (M) axons in the CC of control and Rα*Myrf* ^*fl/fl*^ mice at P57 + 345. [Two‐way mixed effects ANOVA, axon × genotype interaction: F(1,5) = 0.2506; *p* = 0.638; axon F(1,5) = 111.7; *p* < 0.001; genotype: F(1,5) = 1.581, *p* = 0.264]. Graphs show mean ± SD, ^#^ *p* ≤ 0.08, *p < 0.05, **p < 0.01, ***p < 0.001, ****p < 0.0001 by Sidak post‐test. See also Figure S3.

As myelin laid down prior to P57 remains intact in Rα*Myrf* ^*fl/fl*^ mice, acutely preventing adult oligodendrogenesis would not be expected to grossly affect the proportion of callosal axons that are myelinated. However, over a longer period of time, preventing adult oligodendrogenesis should lead to Rα*Myrf* ^*fl/fl*^ mice having fewer myelinated callosal axons than control mice. Our analysis of transmission electron micrographs from the CC of P57 + 60 and P57 + 345 (Figure 5U–X) revealed that total axon density was not affected by age or genotype [P57 + 60 Control, 2.15 ± 0.26 axons/μm^2^; P57 + 60 Rα*Myrf* ^*fl/fl*^, 1.67 ± 0.63 axons/μm^2^; P57 + 345 Control, 1.65 ± 0.32 axons/μm^2^; P57 + 345 Rα*Myrf* ^*fl/fl*^, 2.44 ± 0.34 axons/μm^2^: two‐way mixed effects ANOVA, interaction F(1,9) = 9.208, *p* = 0.014; age F(1,9) = 0.323, *p* = 0.58; genotype: F(1,9) = 0.594, *p* = 0.46; *p* > 0.1 for all Bonferroni corrected multiple comparisons]. Furthermore, myelinated axon g‐ratio was normal in P57 + 60 and P57 + 345 Rα*Myrf* ^*fl/fl*^ mice (Figure S3). As predicted, the proportion of callosal axons that were myelinated was equivalent in P57 + 60 control and Rα*Myrf* ^*fl/fl*^ mice (Figure 5Y). However, at P57 + 345 more CC axons tended to be myelinated in control compared to Rα*Myrf* ^*fl/fl*^ mice (Figure 5Y; Sidak multiple comparison test, *p* = 0.0505). While unmyelinated axon diameter could have increased over time in Rα*Myrf* ^*fl/fl*^ mice, as this process is mechanistically independent of myelination (Bin et al. 2025), we found that unmyelinated and myelinated axon diameters were not affected by genotype (Figure 5Z).

### 3.5 Preventing Adult Oligodendrogenesis Does Not Impair Motor Performance

We next aimed to determine whether new myelin was required to sustain axon‐glial domain integrity or execute established motor behaviors. Unlike genetic approaches that prevent adult oligodendrogenesis by modifying OPC function (Cullen et al. 2020; McKenzie et al. 2014; Schneider et al. 2016), the conditional deletion of *Myrf* from adult OPCs did not impact nodal integrity, action potential conduction velocity, or motor behavior (Figure 6). When we performed immunohistochemistry to detect NaV1.6^+^ nodes and Caspr^+^ paranodes in the CC (Figure 6A–D), we determined that average node (Figure 6E) and paranode lengths (Figure 6F) and node length distributions (Figure 6G,H) were normal in P57 + 60 and P57 + 345 Rα*Myrf* ^*fl/fl*^ mice. Furthermore, the conditional deletion of *Myrf* from adult OPCs was not associated with a rapid or overt change in the callosal CAP (Figure 6I). At P57 + 60, the amplitude (Figure 6J) and half‐width (Figure 6K) of the M axon CAP peak was equivalent for control and Rα*Myrf* ^*fl/fl*^ mice, and M axon CAP conduction velocity was unaffected by genotype (Figure 6L).

**FIGURE 6:**
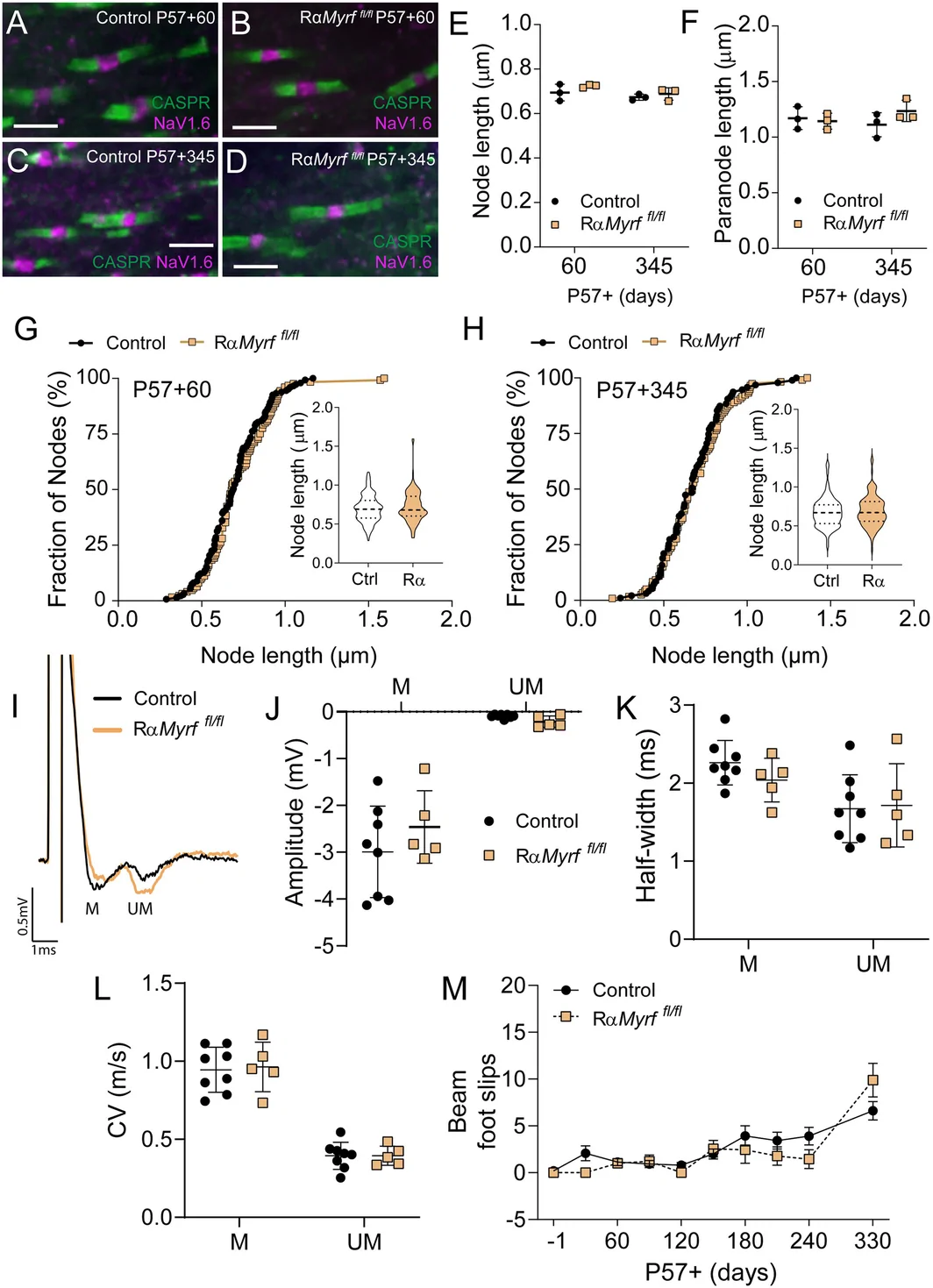
Node and paranode lengths, and callosal CAP conduction is normal in P57 + 60 RαMyrf^*fl/fl*^ mice. (A–D) Single confocal scans showing nodes of Ranvier (Nav1.6, magenta) and paranodes (caspr, green) in the CC of control and Rα*Myrf* ^*fl/fl*^ mice at P57 + 60 and P57 + 345. Scale bar represents 2 μm. (E) Quantification of average node of Ranvier length in the CC of control and Rα*Myrf* ^*fl/fl*^ mice at P57 + 60 and P57 + 345. [Two‐way ANOVA, timepoint*genotype interaction: F(1,8) = 0.276, *p* = 0.613; timepoint: F(1,8) = 3.90, *p* = 0.083; genotype: F(1,8) = 2.416, *p* = 0.1587]. Mean ± SD. (F) Quantification of average paranode length in the CC of control and Rα*Myrf* ^*fl/fl*^ mice at P57 + 60 and P57 + 345. [Two‐way ANOVA, timepoint × genotype interaction: F(1,8) = 1.886, *p* = 0.206; timepoint: F(1,8) = 0.092, *p* = 0.76; genotype: F(1,8) = 0.766, *p* = 0.406]. Mean ± SD. (G) Node of Ranvier length distribution in the CC of control (*n* = 147 nodes) and Rα*Myrf* ^*fl/fl*^ (*n* = 125 nodes) mice at P57 + 60. Kolmogorov–Smirnov test, K‐S D = 0.087, *p* = 0.68; Mann–Whitney U test (inset), M‐W U = 8596, *p* = 0.36. Median (solid line) ± interquartile range (dashed lines). (H) Node of Ranvier length distribution in the CC of control (*n* = 95 nodes) and Rα*Myrf* ^*fl/fl*^ (*n* = 119 nodes) mice at P57 + 345. Kolmogorov–Smirnov test, K‐S D = 0.087, *p* = 0.81; Mann–Whitney U test (inset), M‐W U = 5370, *p* = 0.53. Median (solid line) ± interquartile range (dashed lines). (I) Example callosal CAP traces from control and Rα*Myrf* ^*fl/fl*^ mice at P57 + 60. (J) Amplitude of the myelinated (M) and unmyelinated (UM) peaks of the callosal CAPS from control and Rα*Myrf* ^*fl/fl*^ mice at P57 + 60. [Two‐way ANOVA, axon × genotype interaction: F(1,22) = 1.557, *p* = 0.225; axon population: F(1,22) = 97.98, *p* < 0.0001; genotype: F(1,22) = 0.634, *p* = 0.43]. Mean ± SD. (K) Half‐width of the M and UM peaks of the callosal CAPS from control and Rα*Myrf* ^*fl/fl*^ mice at P57 + 60. [Two‐way ANOVA, axon × genotype interaction: F(1,22) = 0.035, *p* = 0.85; axon population: F(1,22) = 134.8, *p* < 0.0001; genotype: F(1,22) = 0.035, *p* = 0.85]. Mean ± SD. (L) Velocity of the M and UM axons in the callosal CAPs of control and Rα*Myrf* ^*fl/fl*^ mice at P57 + 60. [Two‐way ANOVA, axon × genotype interaction: F(1,22) = 0.709, *p* = 0.408; axon population: F(1,22) = 8.452, *p* = 0.008; genotype: F(1,22) = 0.322, *p* = 0.57]. Mean ± SD. (M) Quantification of foot slips made by control and Rα*Myrf* ^*fl/fl*^ mice performing the beam walk test from P57 − 1 to P57 + 330. [Restricted Maximum Likelihood (REML) mixed effects model: F(1,22) = 0.749, *p* = 0.396; age F(3.776,79.71) = 19.76, *p* < 0.0001; interaction F(9,190) = 2.445, *p* = 0.012]. Mean ± SEM. See also Figure S4.

To determine whether adult‐born OLs and myelination influence movement, balance, or motor coordination, we compared the performance of control and Rα*Myrf* ^*fl/fl*^ mice across a battery of motor function tests, mapping their performance from P57–1, 1 day before tamoxifen delivery, until P57 + 300 or P57 + 330. Consistent with a previous report (McKenzie et al. 2014), latency to fall from the Rotarod was equivalent for control and Rα*Myrf* ^*fl/fl*^ mice at all timepoints examined (Figure S4). Grid‐walk task performance was equivalent for control and Rα*Myrf* ^*fl/fl*^ mice (Figure S4). In the beam‐crossing task, control and Rα*Myrf* ^*fl/fl*^ mice experienced a similar age‐related increase in foot slips, but performance was not impacted by genotype (Figure 6M). From P57–1 to P57 + 330, Digigait stride parameters were largely equivalent between control and Rα*Myrf* ^*fl/fl*^ mice (Figure S4), and Rα*Myrf* ^*fl/fl*^ mice did not experience muscle weakness, as their grip strength measures for fore paws or all paws were equivalent to control mice (Figure S4). At P57 + 60, Rα*Myrf* ^*fl/fl*^ performance in the radial arm maze was also normal (Figure S4). These data suggest that new myelin is not required for mice to continue to perform basic motor tasks or learn in adulthood.

### 3.6 Adult Oligodendrogenesis Promotes Spine Stabilization on the Basal Dendrites of Layer V Pyramidal Neurons

To determine whether new OLs influence synaptic plasticity in adulthood, even under standard housing conditions, we crossed the Rα*Myrf* ^*fl/fl*^ with *Thy1‐YFPH* transgenic mice. This allowed us to visualize and reconstruct the apical (Figure 7A) and basal (Figure 7B) dendrites of layer V pyramidal neurons in P57 + 60 control and Rα*Myrf* ^*fl/fl*^ mice. At P57 + 60, spine density along apical and basal dendrites was unaffected by genotype (Figure 7C). Furthermore, along apical dendrites, the densities of thin, mushroom, and stubby spines (Figure 7D), and the proportion of spines that were each type (Figure 7E), were unchanged between control and Rα*Myrf* ^*fl/fl*^ mice. By contrast, we found that the density of thin spines was significantly elevated along the basal dendrites of P57 + 60 Rα*Myrf* ^*fl/fl*^ mice compared to controls (Figure 7F). As total spine density was normal (Figure 7C), this changed the ratio of thin to mushroom spines. P57 + 60, *RαMyrf* ^*fl/fl*^ mice had a significantly higher proportion of thin spines, and a significantly reduced proportion of mushroom spines, compared to age‐matched controls (Figure 7G).

**FIGURE 7:**
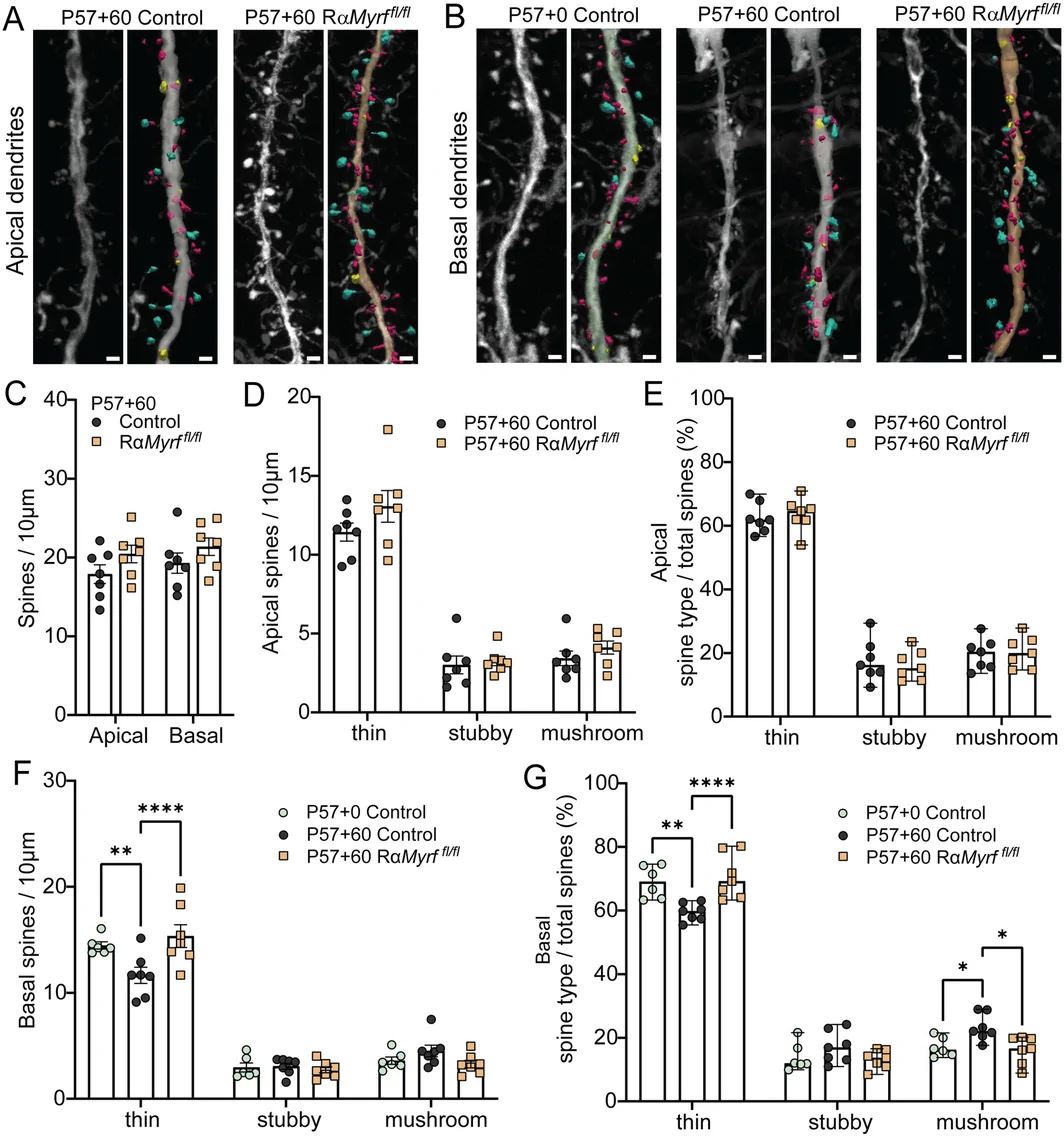
Adult oligodendrogenesis reduces the density of plastic thin spines along the basal dendrites of layer V pyramidal neurons. (A) Representative images of a *Thy1‐YFP* ^+^ layer V MC pyramidal neuron apical dendrite (left panels) and rendered Neurolucida‐360 traced segments (right panels) from P57 + 60 control and Rα*Myrf* ^*fl/fl*^ mice. Spine subtype classifications = thin (magenta); stubby (yellow); mushroom (cyan). (B) Representative images of *Thy1‐YFP* ^+^ layer V MC pyramidal neuron basal dendrites (left panels) and rendered Neurolucida‐360 traced segments (right panels) from P57 + 0 control, P57 + 60 control, and P57 + 60 Rα*Myrf* ^*fl/fl*^ mice. (C) Mean spine densities (number spines per 10 μm dendrite length) for apical and basal dendrites in P57 + 60 control and Rα*Myrf* ^*fl/fl*^ mice [Two‐way mixed ANOVA: Dendrite × group interaction F(1,12) = 0.055 *p* = 0.81, dendrite F(1,12) = 1.372 *p* = 0.26, group F(1,12) = 3.043 *p* = 0.107]. (D) Mean densities of each spine type (thin, mushroom, stubby) along apical dendrites in P57 + 60 control and Rα*Myrf* ^*fl/fl*^ mice [Two‐way mixed ANOVA: Spine type × group interaction F(2,24) = 0.799 *p* = 0.46, spine type F(2,24) = 166.1 *p* < 0.001, group F(1,12) = 2.537 *p* = 0.137] (error bars = SEM). (E) Median percent of spines in each morphological classification (thin, mushroom, stubby) per apical dendrite (95% CIs). The percent of spines in each classification per analyzed apical dendrite segment was not significantly different between genotypes [MWU tests to compare medians, P57 + 60 control versus P57 + 60 Rα*Myrf* ^*fl/fl*^ in each type, all Bonferroni‐corrected *p*‐values > 0.05]. (F) Mean basal dendritic spine densities of each morphological spine type (thin, mushroom, stubby) for P57 + 0 control (green circles); P57 + 60 control (black circles), and P57 + 60 Rα*Myrf* ^*fl/fl*^ (orange squares) mice [Two‐way mixed ANOVA with group (between‐subjects), and spine type (within‐subjects: Thin, mushroom, stubby); type × group interaction: F(4,34) = 6.384, *p* = 0.001; group: F(2,17) = 1.209, *p* = 0.323; type: F(2,34) = 360.02, *p* < 0.001] (error bars = SEM). (G) Median proportion of spines of each morphological type (thin, mushroom, stubby) per basal dendrite (95% CIs) of P57 + 0 control (green circles); P57 + 60 control (black circles), P57 + 60 Rα*Myrf* ^*fl/fl*^ (orange squares) mice [thin spines KW: *H*(2,20) = 13.08, *p* = 0.001; P57 + 60 control versus P57 + 60 Rα*Myrf* ^*fl/fl*^ mice (*z* = −3.30, Bonferroni‐adjusted *p* = 0.003) and P57 + 0 control versus P57 + 60 Rα*Myrf* ^*fl/fl*^ mice (*z* = −2.89, Bonferroni‐adjusted *p* = 0.012); mushroom spines [KW: *H*(2,20) = 9.81, *p* = 0.007], with P57 + 0 controls versus P57 + 60 controls (*z* = 2.58, *p* = 0.03), P57 + 60 control versus P57 + 60 Rα*Myrf* ^*fl/fl*^ (*z* = 2.8, *p* = 0.015), P57 + 0 versus P57 + 60 Rα*Myrf* ^*fl/fl*^ (all *p*‐values > 0.99)]. The percentage of stubby type spines did not differ significantly between groups (*p* > 0.05). Data points represent individual subjects (mean of ≥ 5 dendrite segments per mouse). Scale bars = 1 μm. *p < 0.05, **p < 0.01, ***p < 0.001 denotes Bonferroni‐adjusted *p*‐values.

To determine whether a proportion of thin spines normally mature, stabilise, and accumulate as mushroom spines over time in control mice, and this process fails in Rα*Myrf* ^*fl/fl*^ mice, or mushroom spines are lost over time in Rα*Myrf* ^*fl/fl*^ mice, we imaged and reconstructed the basal dendrites of P57 + 0 *Thy1‐YFPH* control mice. We report that total basal dendritic spine density did not change between P57 + 0 and P57 + 60 [One‐way ANOVA F(2,17) = 1.14, *p* = 0.34; P57 + 0 control mice had 21.02 ± 1.2 spines/10 μm; P57 + 60 control mice had 19.28 ± 3.41 spines/10 μm; P57 + 60 Rα*Myrf* ^*fl/fl*^ mice had 21.36 ± 2.93 spines/10 μm]. However, P57 + 0 control basal dendrites had a higher density of thin spines than the P57 + 60 controls, but resembled the P57 + 60 Rα*Myrf* ^*fl/fl*^ basal dendrites in composition (Figure 7F). Conversely, a lower proportion of P57 + 0 control basal dendritic spines were stable mushroom spines, relative to P57 + 60 controls, but the proportion was equivalent to that of P57 + 60 Rα*Myrf* ^*fl/fl*^ basal dendrites (Figure 7G). These data indicate that new myelin addition is required for layer V MC neurons to increase their proportion of stable mushroom spines across adulthood.

## 4 Discussion

The addition of new myelin to the adult brain supports the acquisition of motor skills (McKenzie et al. 2014; Xiao et al. 2016) and long‐term memory recall (Pan et al. 2020; Steadman et al. 2020). However, it is unclear whether the ability of mature OLs to maintain myelin or the addition of new myelin impacts the strength and stability of synaptic connections. By deleting *Myrf* from mature OLs at P57, we disrupted the transcriptional program that supports OL and myelin maintenance, ultimately inducing CNS demyelination. Within 60 days, MBP levels were reduced, CAP conduction slowed (Figure 1), and PLP*Myrf* ^*fl/fl*^ mice developed fine and gross motor dysfunction that impaired their ability to execute basic as well as established, complex movements (Figure 2). This was accompanied by changes in the postsynaptic structure of layer V MC pyramidal neurons, and their response to glutamate. Apical and basal dendritic spine densities were unchanged, but each dendritic compartment had an increased proportion of spines that were stable, mushroom spines (Figure 3), and mEPSC amplitude was increased (Figure 4). The significant skewing of spine morphology towards mushroom spines, suggests that the OL and myelin maintenance are critical for neurons to sustain a majority population of highly plastic thin or “learning” spines required for neurons to remain adaptable throughout life. Conversely, when *Myrf* was instead deleted from adult OPCs, preventing new OL maturation and further myelin addition (Figure 5), CAP conduction velocity was normal, and the mice were still able to execute basic as well as established, coordinated motor tasks (Figure 6). However, 2 months without adult myelination was sufficient to alter the structure of layer V pyramidal neurons in the MC. In particular, the basal dendrites of P57 + 60 Rα*Myrf* ^*fl/fl*^ retained the higher proportion of thin, transient spines, that was characteristic of P57 + 0 but not P57 + 60 control dendrites, suggesting that the basal dendritic spine configuration was “frozen” at the time of *Myrf* deletion. These data indicate that adult myelination supports the potentiation and stabilization of basal dendritic spines over time, consistent with a role in consolidation.

### 4.1 OL and Myelin Maintenance Allow Layer V Pyramidal Neurons to Balance Synaptic Plasticity and Stability

At P57 + 60, the apical and basal dendrites of PLP*Myrf* ^*fl/fl*^ mice had an elevated proportion of large mushroom spines (Figure 3). The size of the postsynaptic spine head predicts synapse stability, with larger spines having a reduced likelihood of elimination (Holtmaat et al. 2005; Quinn et al. 2019). The larger mushroom spine shape reflects the effect of synaptic potentiation on morphology, particularly the consequence of increased NMDA receptor activity, which increases the density of AMPA receptors at the postsynaptic density (Matsuzaki et al. 2004; Shepherd and Huganir 2007). Therefore, it is likely that layer V pyramidal neurons in PLP*Myrf* ^*fl/fl*^ mice generate larger amplitude mEPSCs than those of control mice (Figure 4), because a higher proportion of their dendritic spines are mushroom spines, with a higher density of AMPA receptors. However, a more direct measurement of AMPA and NMDA receptor currents, including an evaluation of the AMPA/NMDA ratio, would be required to directly evaluate changes in postsynaptic receptor composition. The increased neuronal response to glutamate may explain previous reports that myelin loss is associated with an increase in c‐Fos expression by excitatory neurons in MC (Nguyen et al. 2024) and an increase in sEPSC generation in the striatal medium spiny neurons (Guadalupi et al. 2024), which are synaptic targets of the excitatory layer V MC pyramidal neurons (Hwang et al. 2022).

A larger number of stable mushroom spines could suggest a change in Hebbian or homeostatic plasticity (Hobbiss et al. 2018). For example, demyelination could increase the activity of specific excitatory inputs and strengthen those spines. Alternatively, if the conditional deletion of *Myrf* from OLs produced a sustained decrease in network activity that reduced layer V pyramidal neuron firing, this would trigger a homeostatic response that would increase AMPA receptor expression at the dendritic spines (increasing spine size) to restore neuronal firing (Hobbiss et al. 2018). It is also possible that preventing myelin maintenance could change the expression of neuromodulators like norepinephrine or dopamine, which can change the threshold for long‐term potentiation (Guo et al. 2015; Hu et al. 2007). Irrespective of the cause, the conditional deletion of *Myrf* from OLs produced a major structural and functional change to the major output neurons of the MC that increased their responsiveness to glutamate. Such changes could contribute to neurodegeneration in demyelinating diseases like multiple sclerosis (MS) (Macrez et al. 2016).

While dendritic spine morphology was significantly altered in PLP*Myrf* ^*fl/fl*^ mice, spine density was preserved (Figure 3). By contrast, synapse loss has been reported in the brain, spinal cord, and retina of people with MS prior to neuron loss (Cordano et al. 2024; Dutta et al. 2011; Huiskamp et al. 2022; Jurgens et al. 2016; Mock et al. 2021; Werneburg et al. 2020). Synapse loss has also been reported in rodent models of inflammatory demyelination (Werneburg et al. 2020); however, the outcome is highly variable, with some neurons maintaining synapse density and others even increasing synapse density (Gillani et al. 2024; Potter et al. 2016). It is likely that myelin loss is not the primary driver of synapse loss in demyelinating disease, as data from us and others (Baltan et al. 2021; Lohrberg et al. 2025; Werneburg et al. 2020) suggest that myelin loss can stabilize dendritic spines, reducing the likelihood of spine removal (Quinn et al. 2019). However, changes in spine density may be driven by other elements of the inflammatory environment. For example, in a demyelinating disease context, microglial synapse engulfment would be enhanced by blood‐brain barrier dysfunction and elevated complement C3 expression (Merlini et al. 2019; Werneburg et al. 2020).

### 4.2 Adult Myelination Supports the Stabilization of a Small Number of Synapses Over Time

Preventing OL addition between P57 and P57 + 60 changed the morphological composition of spines along the basal dendrites of layer V pyramidal neurons in the MC. More specifically, P57 + 60 Rα*Myrf* ^*fl/fl*^ mice had dendrites that resembled P57 + 0 rather than P57 + 60 control mice, as they retained a higher proportion of immature, transient, thin spines (Figure 7). This is reminiscent of a previous report showing that without myelin addition from development (P10) through to adulthood, pyramidal neurons in the visual cortex retained their adolescent‐level of plasticity (Xin et al. 2024). Spine structural plasticity is heavily influenced by developmental stage, being less restricted in juvenile than adult mice (Grutzendler et al. 2002; Holtmaat et al. 2005; Trachtenberg et al. 2002; Zuo et al. 2005). This is reflected by an increased proportion of spines having a stable mushroom morphology with age. Live imaging of the apical tufts of layer II/III and V dendrites in the mouse somatosensory cortex revealed that the proportion of stable spines increased from ~35% at P25 to ~54% at P80, ~66% at P120, and ~73% at P225 (Holtmaat et al. 2005). Our data indicate that adult myelination promotes spine stabilization over the lifespan but also show that it exerts a greater influence over spine stabilization in the basal rather than apical dendritic compartment, which may be functionally significant.

Layer V MC pyramidal neurons receive monosynaptic inputs from layers I, II/II, and V M1 neurons, as well as neurons in the frontal association, motor and somatosensory cortices, thalamus, basal ganglia, and other subcortical regions (Geng et al. 2022; Munoz‐Castaneda et al. 2021). From each region, the neurons that synapse with the apical or basal dendrites of layer V MC neurons are almost discrete populations (Geng et al. 2022). The stabilization of new spines on apical dendrites correlates with motor task performance, and their artificial elimination impairs performance of the learned motor task (Hayashi‐Takagi et al. 2015). However, the specific role of basal inputs to layer V MC neurons during task acquisition versus recall requires further investigation. A recent study found that spines along the apical and basal dendrites of layer II/III MC pyramidal neurons applied distinct activity‐dependent plasticity rules (Wright et al. 2025). The strengthening of apical synapses was predicted by their coactivity with other local synapses, while the strengthening of basal dendrites was predicted by their activity being coincident with the postsynaptic action potential (Klionsky et al. 2016; Wright et al. 2025). If layer V MC neurons apply similar compartment‐specific functional synaptic organization, our data could indicate that adult myelination increases the likelihood that basal synapse activity will be coincident with the postsynaptic action potential, to support the transition of select inputs from transient thin to stable mushroom spines over time. While this is not a learning study, synaptic plasticity on layer V pyramidal neuron dendrites plays a crucial role in the acquisition of new motor skills (Albarran et al. 2021; Rioult‐Pedotti et al. 2000; Roth et al. 2020; Xu et al. 2009), and the modulation of synaptic plasticity in discrete dendritic compartments may explain the ability of adult myelination to support learning and memory functions (McKenzie et al. 2014; Pan et al. 2020; Steadman et al. 2020).

## Author Contributions

K.M.Y., C.L.C., B.E., and R.E.P. conceived the project. K.M.Y., C.L.C., B.E., and K.M. acquired funding. B.E. and C.A.B. provided resources. C.A.B., R.E.P., C.L.C., K.M.Y., and K.M. developed methodology. R.E.P., K.M., K.A.P., R.R., P.T.N., C.L.C., and K.M.Y. carried out the experimental investigation. R.E.P., K.M., and C.L.C. were involved in data curation. R.E.P., K.M., C.L.C., A.N., and K.M.Y. conducted formal analysis and visualized the data. K.M.Y. undertook project administration. K.M.Y. and C.L.C. provided supervision and wrote the manuscript.

## Funding

This work was supported by the National Health and Medical Research Council (1030939, 1045240, 1077792, 2012140), Australian Research Council (DP180101494, DP220100100), Medical Research Future Fund (EPCD08), Multiple Sclerosis Australia (11‐014, 15‐054, 16‐105, 17‐0223, 19‐0696, 20‐460, 21‐4‐017), Menzies Institute for Medical Research, Mater Foundation, and Equity Trustees and the Trusts of L G McCallum Est.

## Conflicts of Interest

The authors declare no conflicts of interest.

## Supporting information


**Figure S1:** The conditional deletion of Myrf from OLs at ~2 months of age alters running gait.
**Figure S2:** Small diameter axons appear more vulnerable to myelin loss following *Myrf* deletion from OLs.
**Figure S3:** Conditionally deleting Myrf from OPCs at ~2 months of age does not alter OPC density or myelin thickness on callosal axons.
**Figure S4:** Motor function remains largely normal in RαMyrf^*fl/fl*^ mice.

**Table S1:** Resource Table.

## Data Availability

The data that support the findings of this study are available from the corresponding author upon reasonable request.
