# Supplementary material for: Myelin Maintenance and Addition Regulate Synaptic Plasticity in the Adult Mouse Cortex
Source: Glia. 2026 Oct 11;74(12):e70239. doi: 10.1002/glia.70239 (PMC13655357; doi:10.1002/glia.70239)
Supplement: Supplementary file 1 [file GLIA-74-0-s001.docx]

**Supplementary Table 1: Resource Table**

| REAGENT or RESOURCE | SOURCE | IDENTIFIER |
| --- | --- | --- |
| Antibodies | | |
| Rat (IgG2a) monoclonal (GF090R) anti-GFP (1:2000) | Nacalai Tesque, inc. | Cat# 04404-84; RRID: AB_10013361 |
| Rat monoclonal anti-MBP (1:100) | Abcam | Cat# ab7349; RRID: AB_305969 |
| Mouse (IgG1) monoclonal anti-CASPR (Clone K65/35) (1:200) | UC Davis/NIH NeuroMab Facility | Cat# 75-001; RRID: AB_2083496 |
| Goat (IgG) polyclonal anti-mouse PDGFRα (1:100) | R & D Systems | Cat# AF1062; RRID: AB_2236897 |
| Rabbit polyclonal anti-OLIG2 (1:400) | Millipore | Cat# AB9610; RRID: AB_570666 |
| Rabbit polyclonal anti-ASPA/Nur7 (1:200) | Millipore | Cat# AB1698; RRID: AB_2827931 |
| Rabbit polyclonal anti-Nav1.6 (1:500) | Alamone labs | Cat# ASC-009; RRID: AB_2040202 |
| Rabbit polyclonal anti-GFAP (1:200) | Dako (Aligent) | Cat# Z0334; RRID: AB_10013382 |
| Guinea-pig monoclonal anti-IBA1 (1:200) | Synaptic systems GmbH | Cat# 234004; RRID: AB_2493179 |
| Donkey anti-rat Alexa Fluor _488 (1:500) | Thermo Fisher Scientific | Cat# A-21208; RRID: AB_2535794 |
| Donkey anti-mouse Alexa Fluor-568 (1:1000) | Thermo Fisher Scientific | Cat# A10037; RRID: AB_2534013 |
| Donkey anti-goat Alexa Fluor-568 (1:1000) | Thermo Fisher Scientific | Cat# A-11057; RRID: AB_2534104 |
| Donkey anti-rabbit Alexa Fluor _647 (1:1000) | Thermo Fisher Scientific | Cat# A-31573; RRID: AB_2536183 |
| Hoechst 33342 (1:1000) | Invitrogen | Cat# H1399; RRID: n/a |
| Deposited data | | |
|  |  |  |
| Experimental models: Organisms/strains | | |
| Mouse / C57BL/6J | The Jackson Laboratories | Cat# JAX:000664; RRID: IMSR_JAX:000664 |
| Mouse / Plp-CreER^T2^ | The Jackson Laboratories | Cat# JAX:005975; RRID:  IMSR_JAX:005975 |
| Mouse / Myrf floxed | The Jackson Laboratories | Cat# JAX:010607; RRID:  IMSR_JAX:010607 |
| Mouse / Pdgfra-CreER^TM^ | The Jackson Laboratories | Cat# JAX:018280; RRID:  IMSR_JAX:018280 |
| Mouse / Rosa26-YFP | The Jackson Laboratories | Cat# JAX:006148; RRID:  IMSR_JAX:006148 |
| Oligonucleotides | | |
| Cre 5’ CAGGT CTCAG GAGCT ATGTC CAATT TACTG ACCGTA | Integrated DNA Technologies | n/a |
| Cre 3’ GGTGT TATAAG CAATCC CCAGAA | Integrated DNA Technologies | n/a |
| Rosa26 wildtype 5’ AAAGT CGCTC TGAGT TGTTAT | Integrated DNA Technologies | n/a |
| Rosa26 wildtype 3’ GGAGC GGGAG AAATG GATATG | Integrated DNA Technologies | n/a |
| Rosa26 YFP 50 GCGAA GAGTT TGTCC TCAACC | Integrated DNA Technologies | n/a |
| Myrf 5’ AGGAG TGTTG TGGGA AGTGG | Integrated DNA Technologies | n/a |
| Myrf 3’ CCCAG GCTGA AGATG GAATA | Integrated DNA Technologies | n/a |
| Software and algorithms | | |
| Fiji (ImageJ) | https://imagej.nih.gov/ij/ | RRID: SCR_002285 |
| GraphPad Prism (Versions 9 & 10) | GraphPad Software, Inc. | RRID: SCR_002798 |
| pClamp (version 11) | Molecular Devices | RRID: SCR_011323 |
| Neurolucida 360 | MBF Biosciences LLC | RRID:SCR_016788 |
|  |  |  |
| Other | | |
| Scientifica SliceScope Pro 1000 electrophysiology rig | Scientifica Ltd. | n/a |
| Axopatch 200B patch clamp amplifier | Axon Instruments | n/a |
| Iso-Stim 01D stimulus isolator | NPI Electronic | n/a |
| DigiGaitTM gait analysis system | Mouse Specifics, Inc. | n/a |
| Multi-Maze modular system (RAM) | Ugo Basile | Cat# 41500 |
