# Supplementary material for: Myelin Maintenance and Addition Regulate Synaptic Plasticity in the Adult Mouse Cortex
Source: Glia. 2026 Oct 11;74(12):e70239. doi: 10.1002/glia.70239 (PMC13655357; doi:10.1002/glia.70239)
Supplement: Supplementary file 2 [file GLIA-74-0-s002.docx]

**Supplementary Figures for Pepper, Makowiecki et al.**

**Figure S1:** The conditional deletion of Myrf from OLs at ~2 months of age alters running gait.

A-C) Average forelimb propel time (A), stance time (B) and stride time (C) during one full step by control (black circles) and PLP*Myrf  ^fl/fl^* (purple squares) mice during treadmill (DigiGait^TM^) running from P57-1 to P57+42. [Restricted Maximum Likelihood (REML) mixed effects model: *Propel time* (Interaction F(6, 53)=1.602 p=0.16; day F(6, 53)=1.58 p=0.17; genotype F(1,13)=2.48 p=0.13); *Stance time* (Interaction F(6, 53)=2.48 p=0.034; day F(2.738, 24.18)=1.328 p=0.28; genotype F(1,13)=3.995 p=0.067); *Stride time* (Interaction F(6, 53)=4.87 p=0.0005; day F(2.274, 20.09)=2.121 p=0.14; genotype F(1,13)=3.933 p=0.068)]

D) Average forelimb stride length during one full step by control (black circles) and PLP*Myrf  ^fl/fl^* (purple squares) mice during treadmill (DigiGait^TM^) running from P57-1 to P57+42. [Restricted Maximum Likelihood (REML) mixed effects model: Interaction F(6, 53)=4.91 p=0.0005; day F(2.316, 20.46)=2.076 p=0.14; genotype F(1,13)=3.855 p=0.071]

E-G) Average hindlimb propel time (E), stance time (F) and stride time (G) during one full step by control (black circles) and PLP*Myrf  ^fl/fl^* (purple squares) mice during treadmill (DigiGait^TM^) running from P57-1 to P57+42. [Restricted Maximum Likelihood (REML) mixed effects model: *Propel time* (Interaction F(6, 53)=3.099 p=0.011; day F(2.422, 21.40)=3.07 p=0.058; genotype F(1,13)=2.84 p=0.11); *Stance time* (Interaction F(6, 53)=3.321 p=0.007; day F(3.328, 29.40)=1.449 p=0.24; genotype F(1,13)=7.68 p=0.015); *Stride time* (Interaction F(6, 53)=5.103 p=0.0003; day F(2.775, 24.51)=1.931 p=0.15; genotype F(1,13)=5.328 p=0.038)]

H) Average hindlimb stride length during one full step by control (black circles) and PLP*Myrf  ^fl/fl^* (purple squares) mice during treadmill (DigiGait^TM^) running from P57-1 to P57+42. [Restricted Maximum Likelihood (REML) mixed effects model: Interaction F(6, 53)=5.311 p=0.0002; day F(2.697, 23.82)=1.90 p=0.16; genotype F(1,13)=5.285 p=0.038]

Graphs show mean ± SEM. Control n= 6-9; PLPMyrf *^fl/fl^* n= 4-7. See also Figure 2

**Figure S2:** Small diameter axons appear more vulnerable to myelin loss following *Myrf* deletion from OLs.

A) Quantification of the proportion of area containing MBP+ pixels in the MC and CC of control (n=3; black circles) and PLP*Myrf  ^fl/fl^*  (n=3; purple squares) mice at P57+28. [Two-way ANOVA: Interaction F(1, 8)=4.338, p=0.070; region F(1,8)= 255.2, p=<0.0001; genotype F(1,8)= 15.69, p=0.0042; with Bonferroni post-test]

B) Visualisation of g-ratio vs myelinated axon diameter measurements in the CC of control (n=562 axons; open circles) and PLP*Myrf  ^fl/fl^*  (n=521 axons; purple squares) at P57+35.

C) Quantification of average g-ratio of myelinated axons in the CC of control (n=3; black circles) and PLP*Myrf  ^fl/fl^*  (n=3; purple squares) mice at P57+35 [Unpaired two-tailed t test: t=1.253, df=4, p=0.278]

D) Quantification of average unmyelinated (UM) and myelinated (M) callosal axon diameter from control (n=3) and PLP*Myrf  ^fl/fl^*  (n=3) mice at P57+35. [Two-way ANOVA: Interaction F(1, 8)=0.6002, p=0.46; region F(1,8)= 62.53, p=<0.0001; genotype F(1,8)= 4.74, p=0.061; with Bonferroni post-test]

E-F) Frequency distribution of unmyelinated (E) and myelinated (F) callosal axon diameter of control (n=1310 UM, n=562 M axons; open circles) and PLP*Myrf  ^fl/fl^*  (n=1679 UM, n=521 M axons; purple squares) at P57+35. [Kolmogorov-Smirnov test (K-S): unmyelinated K-S D= 0.1538; myelinated K-S D= 0.1570; Mann-Whitney U test (inset violin plots): unmyelinated M-W U = 894610, myelinated M-W U = 114142]

Graphs show mean ± SD or Median and interquartile range (violin plots) *p<0.05, **p<0.01, ***p<0.001, ****p<0.0001. See also Figure 1

**Figure S3:** Conditionally deleting Myrf from OPCs at ~2 months of age, does not alter OPC density or myelin thickness on callosal axons.

A-B) Density of PDGFRα+ OPCs in the motor cortex (A) and corpus callosum (B) of control (black circles) and Rα*Myrf ^fl/fl^* (orange squares) mice at P57+30 to P57+345 days. Two-way ANOVA: *motor cortex* Interaction F(4, 21)=0.544 p=0.704; day F(4, 21)=3.71 p=0.019; genotype F(1,21)=2.45 p=0.13;  *corpus callosum* Interaction F(4, 23)=0.478 p=0.75; day F(4, 23)=5.23 p=0.003; genotype F(1,23)=7.931 p=0.009]

C) Proportion of recombined OPCs (YFP^+^ PDGFRα^+^) in the corpus callosum of of control (black circles) and Rα*Myrf ^fl/fl^* (orange squares) mice from P57+30 to P57+345. Two-way ANOVA: Interaction F(4, 22)=2.893 p=0.045; day F(4, 22)=9.689 p=0.0001; genotype F(1,22)=0.018 p=0.894

D-E) High magnification Transmission electron micrographs through the CC P57+345 control (D) and Rα*Myrf ^fl/fl^* (E) mice. Scale bars represent 50nm.

F-G) Visualisation of g-ratio vs myelinated axon diameter measurements from the CC of control (open circles) and Rα*Myrf ^fl/fl^* (orange squares) mice at P57+60 (F; n= 234 control, 204 Rα*Myrf ^fl/fl^* axons) and P57+345 (I; n= 502 control, 506 Rα*Myrf ^fl/fl^* axons).

Graphs show mean ± SD, *p<0.05, **p<0.01, ***p<0.001, by Sidak post-test. See also Figure 5.

**Figure S4:** Motor function remains largely normal in RαMyrf ^fl/fl^ mice

A) Time taken for control (black circles, n= 13-58) and Rα*Myrf ^fl/fl^* (orange squares, n= 9-51) mice to fall from the rotarod at P57-1 to P57+330. [Restricted Maximum Likelihood (REML) mixed effects model: Interaction F(15, 722) = 0.488 p=0.94; day F(15, 722) = 3.94 p<0.0001; genotype F(1, 107) = 0.39 p=0.53].

B) Proportion of missteps made by control (n=9-15) and Rα*Myrf ^fl/fl^* (n=7-9) mice performing the grid walk test at P57-1 to P57+330. [Restricted Maximum Likelihood (REML) mixed effects model: Interaction F(9, 173) = 1.417 p=0.18; day F(4.46, 85.75) =3.677 p=0.0063; genotype F(1, 22) = 2.601 p=0.12].

C) Grip strength for all paws and forepaws only of control (n= 13-58) and Rα*Myrf ^fl/fl^* (n= 9-51) mice from P57-1 to P57+330. [Restricted Maximum Likelihood (REML) mixed effects model: *All paws* (Interaction F(15, 778) = 2.818 p= 0.00033; day F(15, 778) = 30.74 p<0.0001; genotype F(1,122) = 3.267 p=0.073); *Fore paws* (Interaction F(15, 758)=0.56 p=0.90; day F(15, 758)=3.469 p<0.0001; genotype F(1,112) = 1.057 p=0.306)]

D-E) Average hindlimb stride frequency (D) and limb swing time (E) of control (n=5-18) and Rα*Myrf ^fl/fl^* (n=7-22) mice during treadmill (DigiGait^TM^) running from P57-1 to P57+330. [Restricted Maximum Likelihood (REML) mixed effects model: *Stride frequency* (Interaction F(11, 186) = 0.599 p=0.82; day F(4.432, 74.94) = 8.489 p<0.0001; genotype F(1, 44) = 0.577 p=0.45); *Swing* (Interaction F(11, 187) = 0.956 p=0.48; day F(4.97, 84.49) = 2.945 p=0.017; genotype F(1,45) = 8.112 p=0.006)]

F) Average hindlimb stride length during one full step by control and Rα*Myrf ^fl/fl^* mice during treadmill (DigiGait^TM^) running from P57-1 to P57+330. [Restricted Maximum Likelihood (REML) mixed effects model: Interaction F(11, 186) = 0.565 p=0.85; day F(4.407, 74.52) = 8.54 p<0.0001; genotype F(1,44) = 1.019 p=0.31]

G-H) Average forelimb stride frequency (G) and limb swing time (H) of control and Rα*Myrf ^fl/fl^* mice during treadmill (DigiGait^TM^) running from P57-1 to P57+330. [Restricted Maximum Likelihood (REML) mixed effects model: *Stride frequency* (Interaction F(11, 186) = 0.849 p=0.59; day F(5.79, 98.04) = 7.84 p<0.0001; genotype F(1,44) = 0.964 p=0.33); *Swing* (Interaction F(11, 186) =1.21 p=0.28; day F(5.32, 89.48) = 3.24 p=0.0085; genotype F(1,44)=3.775 p=0.058)]

I) Average forelimb stride length during one full step by control and Rα*Myrf ^fl/fl^* mice during treadmill (DigiGait^TM^) running from P57-1 to P57+330. [Restricted Maximum Likelihood (REML) mixed effects model: Interaction F(11, 186) = 0.77 p=0.66; day F(4.587, 78.40) = 4.328 p=0.0004; genotype F(1,44) = 1.055 p=0.309]

J) Quantification of the average number of errors made across 3 trials each day by control (n=8) and Rα*Myrf ^fl/fl^* (n=7) mice learning the RAM task from P57 + 60. [RM two-way ANOVA: Interaction F(10, 130)=1.467, p=0.158; day F(4.876, 63.39) = 24.28, p<0.0001; genotype F(1, 13) = 0.06, p=0.807]

Graphs show mean ± SEM.
